# *Shigella flexneri* Adherence Factor Expression in *In Vivo*-Like Conditions

**DOI:** 10.1128/mSphere.00751-19

**Published:** 2019-11-13

**Authors:** Rachael B. Chanin, Kourtney P. Nickerson, Alejandro Llanos-Chea, Jeticia R. Sistrunk, David A. Rasko, Deepak Kumar Vijaya Kumar, John de la Parra, Jared R. Auclair, Jessica Ding, Kelvin Li, Snaha Krishna Dogiparthi, Benjamin J. D. Kusber, Christina S. Faherty

**Affiliations:** a Mucosal Immunology and Biology Research Center, Division of Pediatric Gastroenterology and Nutrition, Massachusetts General Hospital, Boston, Massachusetts, USA; b Department of Pediatrics, Harvard Medical School, Boston, Massachusetts, USA; c Institute for Genome Sciences, Department of Microbiology and Immunology, University of Maryland School of Medicine, Baltimore, Maryland, USA; d Genetics and Aging Research Unit, Department of Neurology, Massachusetts General Hospital, Boston, Massachusetts, USA; e Biopharmaceutical Analysis Training Laboratory, Northeastern University Innovation Campus, Burlington, Massachusetts, USA; University of Iowa

**Keywords:** *Shigella flexneri*, adherence factors, long polar fimbriae, type 1 fimbriae, curli, bile salts, glucose, biofilm, *in vivo*-like conditions, intestinal, epithelial cells

## Abstract

The *Shigella* species are Gram-negative, facultative intracellular pathogens that invade the colonic epithelium and cause significant diarrheal disease. Despite extensive research on the pathogen, a comprehensive understanding of how *Shigella* initiates contact with epithelial cells remains unknown. *Shigella* maintains many of the same Escherichia coli adherence gene operons; however, at least one critical gene component in each operon is currently annotated as a pseudogene in reference genomes. These annotations, coupled with a lack of structures upon microscopic analysis following growth in laboratory media, have led the field to hypothesize that *Shigella* is unable to produce fimbriae or other traditional adherence factors. Nevertheless, our previous analyses have demonstrated that a combination of bile salts and glucose induces both biofilm formation and adherence to colonic epithelial cells. The goal of this study was to perform transcriptomic and genetic analyses to demonstrate that adherence gene operons in Shigella flexneri strain 2457T are functional, despite the gene annotations. Our results demonstrate that at least three structural genes facilitate S. flexneri 2457T adherence for epithelial cell contact and biofilm formation. Furthermore, our results demonstrate that host factors, namely, glucose and bile salts at their physiological concentrations in the small intestine, offer key environmental stimuli required for adherence factor expression in S. flexneri. This research may have a significant impact on *Shigella* vaccine development and further highlights the importance of utilizing *in vivo*-like conditions to study bacterial pathogenesis.

**IMPORTANCE** Bacterial pathogens have evolved to regulate virulence gene expression at critical points in the colonization and infection processes to successfully cause disease. The *Shigella* species infect the epithelial cells lining the colon to result in millions of cases of diarrhea and a significant global health burden. As antibiotic resistance rates increase, understanding the mechanisms of infection is vital to ensure successful vaccine development. Despite significant gains in our understanding of *Shigella* infection, it remains unknown how the bacteria initiate contact with the colonic epithelium. Most pathogens harbor multiple adherence factors to facilitate this process, but *Shigella* was thought to have lost the ability to produce these factors. Interestingly, we have identified conditions that mimic some features of gastrointestinal transit and that enable *Shigella* to express adherence structural genes. This work highlights aspects of genetic regulation for *Shigella* adherence factors and may have a significant impact on future vaccine development.

## Abstract (precis)

Bacterial pathogens have evolved to regulate virulence gene expression at critical points in the colonization and infection processes to successfully cause disease. The *Shigella* species infect the epithelial cells lining the colon to result in millions of cases of diarrhea and a significant global health burden. As antibiotic resistance rates increase, understanding the mechanisms of infection is vital to ensure successful vaccine development. Despite significant gains in our understanding of *Shigella* infection, it remains unknown how the bacteria initiate contact with the colonic epithelium. Most pathogens harbor multiple adherence factors to facilitate this process, but *Shigella* was thought to have lost the ability to produce these factors. Interestingly, we have identified conditions that mimic some features of gastrointestinal transit and that enable *Shigella* to express adherence structural genes. This work highlights aspects of genetic regulation for *Shigella* adherence factors and may have a significant impact on future vaccine development.

## INTRODUCTION

Shigella flexneri is a Gram-negative, facultative anaerobe that infects millions of people each year and that causes watery or bloody diarrhea, cramping, and dehydration. *Shigella* infection is endemic in developing countries, causing significant mortality and morbidity, particularly in children under the age of 5 years (1). In industrialized nations, infection is episodic and primarily linked to contaminated food or water. Infection in nonimmunocompromised individuals is self-limiting, and most patients recover with oral rehydration therapy and antibiotics (2–4). However, the increasing prevalence of antibiotic resistance (5) highlights the need to pursue effective vaccine strategies in these enteric pathogens that are gaining resistance mechanisms.

The current *Shigella* infection paradigm is that the bacteria spread through fecal-oral transmission, in which an extremely low infectious dose, with as few as 10 to 100 organisms, initiates infection (2). Once it is ingested, *Shigella* traverses the digestive tract and localizes to the colon. To invade the colonic epithelium, *Shigella* transits through M (microfold or membranous) cells, which are specialized antigen-presenting cells of the follicle-associated epithelium (FAE) (6). Transit through M cells allows the bacteria to reach the basolateral pole of the epithelium for invasion (2), and the FAE is considered the major site of entry for *Shigella* due to the presence of M cells (7). Following basolateral invasion, intracellular replication, and intercellular spread, polymorphonuclear cells are recruited to the site of infection to eliminate the pathogen. The massive tissue destruction that results in the symptoms of bacillary dysentery is due to this intense inflammatory response (2).

While the invasion process and intracellular spread, replication, and survival of *Shigella* have been thoroughly investigated, much less is known about the virulence dynamics of the bacteria prior to invasion and transcytosis. In fact, there is a critical gap in knowledge regarding how the bacteria target M cells to initiate the invasion process and whether *Shigella* utilizes adherence factors to adhere to the apical surface of epithelial cells prior to invasion. Due to the mucosal environment encountered on the surface of gastrointestinal epithelial cells, many pathogens, particularly pathogenic Escherichia coli and *Salmonella* species, often utilize pili, fimbriae, or afimbrial adhesins to efficiently colonize host cells (8–13). Because *Shigella* and E. coli are closely related (14, 15) and because fimbriae are prevalent among the *Enterobacteriaceae* (16), it is reasonable to hypothesize that *Shigella* utilizes fimbriae or other adhesins during colonization. Interestingly, *Shigella* is thought to have lost the ability to produce traditional E. coli adherence factors as the bacteria adapted to an intracellular lifestyle (2) due to three main reasons. First, *Shigella* strains grown in standard laboratory media lack visible adhesive structures upon transmission electron microscopy (TEM) (17, 18), unlike some strains of E. coli, in which adherence factors are thought to be constitutively expressed (19, 20). Second, examination of *Shigella* genomes deposited in GenBank reveals that almost all adherence gene clusters, such as those for type 1 fimbriae (10, 21) and curli (22), contain at least one annotated pseudogene that is crucial for either the adherence factor structure or the assembly process (17, 23, 24). Third, the production of adherence factors is considered counterproductive to the lifestyle of an intracellular pathogen evading immune detection (2, 25, 26).

Despite this null adherence factor hypothesis, a limited number of reports have detected adherence factor expression in S. flexneri (27–29), but, unfortunately, in-depth genetic analyses have not been performed. Furthermore, we have previously demonstrated that tryptic soy broth (TSB) media supplemented with bile salts induce the adherence of S. flexneri 2457T to colonic epithelial cells, which is facilitated at least in part by the type III secretion system effector proteins OspE1 and OspE2 (30). Finally, our recent publication characterizes an adhesive biofilm phenotype following exposure to a combination of bile salts and glucose that represents aspects of the *in vivo*-like conditions (IVLCs) found in the small intestine (31–36). Given this literature and the fact that deletion of both *ospE1* and *ospE2* did not completely abrogate adherence (30), we sought to determine if additional adherence genes are expressed by S. flexneri 2457T following exposure to IVLCs. In this study, we performed transcriptomic and genetic analyses to begin to characterize the adherence gene clusters in S. flexneri 2457T. Our results demonstrate that at least three structural genes facilitate adherence for both biofilm formation and colonization of colonic epithelial cells, particularly in the human intestinal organoid-derived epithelial monolayer (HIODEM) model. This work broadens our understanding of S. flexneri 2457T pathogenesis and demonstrates that S. flexneri 2457T likely expresses several traditional adherence factors important for pathogenesis. Insights gained from this work could have an important impact on *Shigella*-specific therapeutic and vaccine development.

## RESULTS

### S. flexneri 2457T produces putative adherence structures in IVLCs.

**FIG 1:**
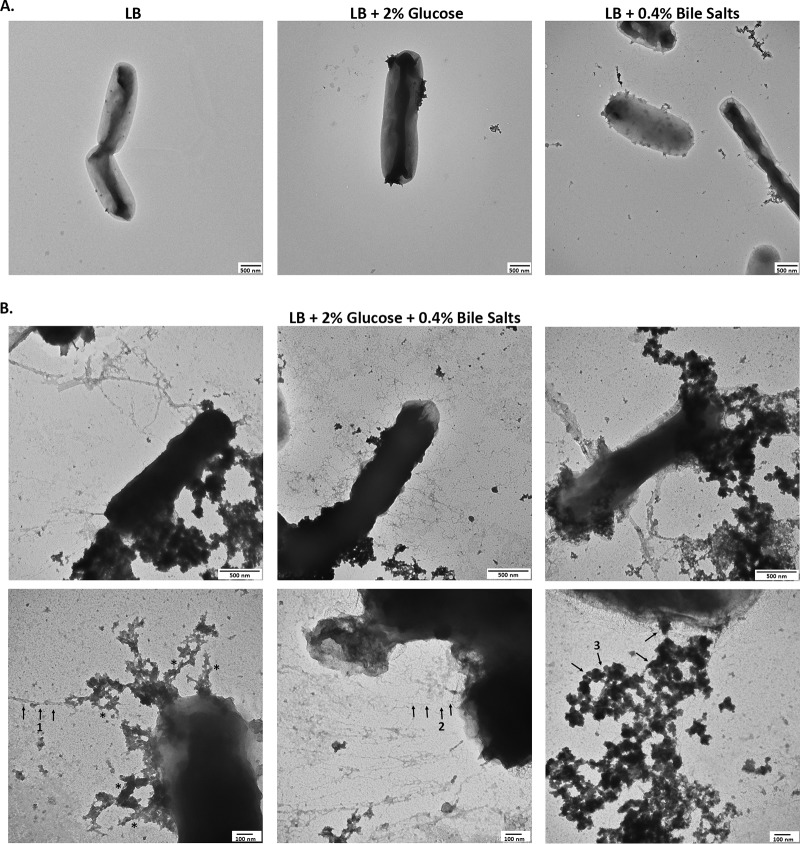
The growth of S. flexneri 2457T in IVLCs produces several putative adherence structures. Cultures of wild-type strain 2457T were grown overnight with static growth in the indicated media. Samples were negatively stained and imaged by electron microscopy. Images are representative of those from at least 3 biological replicates. (A) TEM analysis of 2457T grown in LB, LB supplemented with 2% glucose, or LB supplemented with 0.4% bile salts demonstrated that adherence factors were either not produced or minimally produced under these conditions. Magnification, ×25,000; scale bar, 500 nm. (B) 2457T grown in LB supplemented with both 2% glucose and 0.4% bile salts (IVLCs) revealed three types of putative adherence factors upon TEM analysis. Magnifications, ×50,000 (top row; scale bar, 500 nm) and ×100,000 magnification (bottom row; scale bar, 100 nm). Different structures are highlighted by numbered arrows. Arrow 1 points to thicker structures, arrow 2 points to thinner structures, and arrow 3 points to electron-dense aggregates. The asterisks denote rough, complex structures (refer to Discussion).

**FIG 2:**
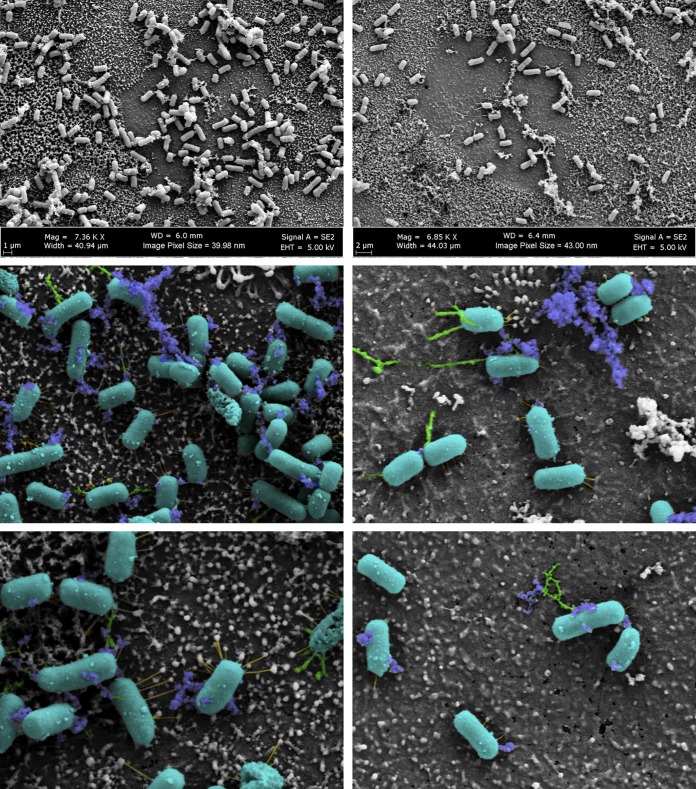
Scanning electron microscopy of S. flexneri 2457T adherence on the human intestinal organoid-derived epithelial monolayer model. S. flexneri 2457T was subcultured in IVLCs, washed, and applied for adherence analysis on the HIODEM model. Following infection, samples were fixed and processed for SEM analysis. The images on the top row display the association of bacteria with the cells of the model. The middle and bottom rows are images from a higher magnification. Magnifications, approximately ×7,000 (top row) and approximately ×20,000 (bottom row). Pseudocoloring was performed to enhance the visualization of the three types of putative adherence factors visualized on the bacteria that interact with the apical surface of the epithelial cells. The bacterial rods are colored teal, thicker structures are colored green, thinner structures are colored yellow, and electron-dense aggregates are colored blue. The images in the left and right columns represent those from two separate biological samples.

Our previous work demonstrated that S. flexneri 2457T grown in IVLCs produced a biofilm. Furthermore, upon bacterial dispersion from the biofilm, recovered bacteria displayed induced adherence to colonic HT-29 cells. This analysis enabled us to expand the *Shigella* infection paradigm to incorporate biofilm formation due to exposure to IVLCs during small intestinal passage, biofilm dispersion upon colonic transition following the loss of the bile salts signal, and the subsequently induced infection (31, 35). Since adherence factors are important components of biofilm formation (35, 37), we performed electron microscopy (EM) analysis of bacteria isolated from the IVLC-induced biofilm to visualize possible adherence factors. As shown in Fig. 1 and in Fig. S1 in the supplemental material, bacteria produced thicker and thinner structures of various lengths and electron-dense aggregates in IVLCs. Bacteria grown in Luria broth (LB) and LB supplemented with glucose (2%, wt/vol) lacked structures, while bacteria grown in LB medium supplemented with bile salts (0.4%) produced very minimal structures. The utilization of bile salts in tryptic soy broth (TSB) medium, in which there is additional glucose relative to the amount in LB medium (31), resulted in the appearance of putative adherence structures similar to those seen in LB medium supplemented with both glucose and bile salts (Fig. S1). The data confirmed our observations that glucose and bile salts (IVLCs) are required for S. flexneri 2457T to form an adhesive biofilm (31). To support the biofilm data and our previous induced HT-29 cell adherence observations (30, 31), we performed adherence analysis on a human intestinal organoid-derived epithelial monolayer (HIODEM) model. The model is derived from stem cells isolated from intestinal tissue, propagated as organoids, and subsequently trypsinized and seeded onto transwells to generate a two-dimensional (2-D) polarized, differentiated model of the intestinal epithelium in which enterocytes, mucus-producing goblet cells, and antigen-sampling M cells are present (38–42). With the model derived from the ascending colon, S. flexneri 2457T subcultured in IVLCs displayed putative adherence structures contacting the epithelial cells (Fig. 2). In all, the data suggest that these putative adherence structures are important for both biofilm formation and adherence to colonic epithelial cells.

10.1128/mSphere.00751-19.1 FIG S1 Additional images. (A) Additional images of strain 2457T grown in LB with 2% glucose and 0.4% bile salts. Further TEM analyses were performed to capture additional phenotypes of putative adherence structures and provide higher-magnification images to demonstrate that the structures originate from the bacterial cells (white arrows). Asterisks mark areas that displayed possible curli fibrils. The images are from experiments biologically independent of those from which the images provided in Fig. 1 and 7 were obtained. Each image displays a separate bacterial cell unless paired with a matching boxed sample. The magnifications and scale bars are provided for each image or image sets. (B) Wild-type cultures of 2457T were grown overnight by static growth in TSB media supplemented with 0.4% (wt/vol) bile salts. TEM analysis revealed structures similar to those of bacteria grown in LB supplemented with 2% glucose and 0.4% bile salts. Magnification, ×50,000; bar, 500 nm. (C) Two images of control media (LB with 2% glucose and 0.4% bile salts) are on top. Samples were prepared and processed as described in the Materials and Methods section for the biofilm assay and subsequent TEM grid preparation. TEM analysis revealed some precipitates of material from either the media or the uranyl acetate stain; however, the precipitation varied in concentration and did not resemble the structures observed in the presence of bacteria. Magnifications, ×50,000; bars, 500 nm (from separate experiments). (D) An image of an area without bacteria present of a TEM grid prepared from the 2457T biofilm (grown in IVLC medium). The same precipitation seen on the control medium grids tended to be less concentrated in clean areas of the bacterial grids. Magnification, ×50,000; bar, 500 nm. Download FIG S1, PDF file, 1.7 MB. Copyright © 2019 Chanin et al. 2019 Chanin et al. This content is distributed under the terms of the Creative Commons Attribution 4.0 International license.

### S. flexneri 2457T maintains and transcribes several adherence gene clusters.

**FIG 3:**
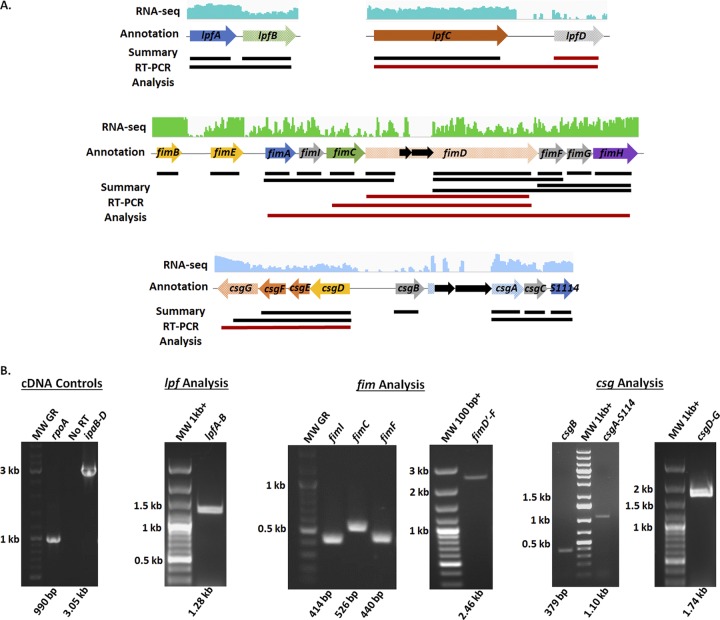
*In silico* and transcriptomic analysis of the S. flexneri 2457T adherence gene clusters. (A) Identification of the long polar fimbriae (*lpf*), type 1 fimbriae (*fim*), and curli (*csg*) adherence gene clusters in the S. flexneri 2457T genome was performed using the NCBI GenBank tool. Arrows represent annotated open reading frames (ORF), in which blue arrows represent the annotated major subunits, green arrows represent the annotated chaperones, orange arrows represent the annotated ushers or pores, yellow arrows represent the annotated regulatory subunits, purple arrows represent the annotated tip adhesins, and gray arrows represent additional putative adherence genes involved in assembly or secretion. All arrows with checkered backgrounds are annotated as pseudogenes in NCBI due to predicted insertion sequences, truncations, or frameshifts. Insertion sequences or transposons are represented by solid black arrows. The RNA sequencing trace read data are provided for each gene cluster above the arrows. The best trace read available is presented, with green representing shaking growth conditions (darker green for growth in bile salts) and blue representing static growth conditions (darker blue for growth in bile salts). The solid lines below the arrows represent a summary of the confirmation of gene transcription that resulted from nonquantitative RT-PCR analyses. Single-gene and polycistronic amplifications were performed to obtain the largest products possible for each operon. Red lines represent products that were not obtained. Refer to Fig. S2 in the supplemental material for the results of additional gene cluster analyses. (B) Representative gel electrophoresis images of nonquantitative RT-PCR analyses of the various adherence gene clusters are provided. Biologically independent RNA samples were used in the analysis, in which RNA integrity was verified by amplifying the housekeeping gene *rpoA* as well as the *ipaB* to *ipaD* operon encoded on the virulence plasmid. Control amplifications without reverse transcriptase in the *rpoA* reaction mixtures ensured that there was no DNA contamination of the RNA samples prior to cDNA synthesis. Each gene is labeled with the expected molecular weight (MW) of the product provided below the gel image. Note that different molecular weight ladders were utilized in the analyses. Refer to the Materials and Methods section for more information.

**FIG 4:**
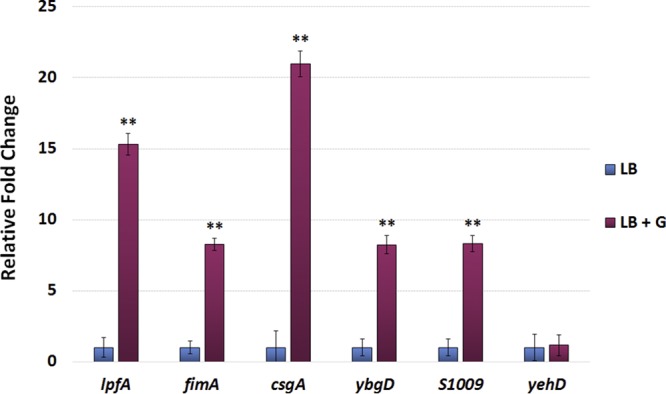
Quantitative RT-PCR analysis of S. flexneri 2457T structural adherence genes. RNA was isolated from S. flexneri 2457T grown in medium (LB) or medium supplemented with 2% (wt/vol) glucose (LB + G). For each primer set, the relative fold change ± the standard error of the Δ*C_T_* value is plotted for each gene. Data represent the average for at least three biological independent samples, in which each sample had technical duplicates. *yehD* (Table 1) expression was not induced in the presence of glucose and therefore served as an internal negative control for the analysis. **, *P* ≤ 0.01 between LB and LB-glucose conditions.

We next examined the transcription of the S. flexneri 2457T adherence genes under various conditions. *In silico* analyses of the annotated S. flexneri 2457T genome in the NCBI GenBank database identified several adherence gene components (Table 1, Fig. 3, and Fig. S2). These genes are maintained in S. flexneri 2457T, despite examples of full gene and/or operon deletions for some of the adherence gene clusters in other *Shigella* species (23). As documented in previous studies (17, 23, 24), all S. flexneri 2457T adherence gene clusters contain at least one annotated pseudogene (due to predicted point mutations, truncations, or insertion sequences), supporting hypotheses stating that *Shigella* cannot produce traditional adherence factors. However, our previous RNA sequencing (RNA-seq) data (31) indicated that despite the gene annotations, most of the adherence genes were transcribed by S. flexneri 2457T (Fig. 3 and Fig. S2). To confirm the RNA-seq results, we performed reverse transcription-PCR (RT-PCR) analysis of the annotated adherence gene clusters (Fig. 3 and Fig. S2). RNA isolated from S. flexneri 2457T broth cultures were positive for transcription of the adherence genes and large segments of the predicted operons. Insertion sequences did not prevent the transcription of large downstream segments. For example, as demonstrated in Fig. 3, we amplified cDNA products from *fimD* just after the insertion sequences to the end of *fimF*. Finally, we utilized quantitative RT-PCR (qRT-PCR) analysis to obtain additional data to support the transcription of adherence genes. As described in previously published literature for other pathogens (43–46), glucose induced the expression of the S. flexneri 2457T genes encoding structural subunits (Fig. 4). In all, the data indicate that adherence gene clusters are genomically maintained and transcriptionally regulated in S. flexneri 2457T, despite the pseudogene annotations.

**TABLE 1:** Adherence gene clusters identified in the Shigella flexneri 2457T genome^*a*^

Adherence gene	Locus tag	Annotated function/pseudogene
Long polar fimbriae		
***lpfA***	**S3961**	**Major fimbrial subunit**
*lpfB*		Chaperone/pseudogene
*lpfC*	S4048	Outer membrane usher
*lpfD*		Fimbrial protein/pseudogene
Type 1 fimbriae		
*fimB*	S4467	Recombinase
*fimE*	S4466	Tyrosine recombinase
***fimA***	**S4465**	**Major fimbrial subunit**
*fimI*	S4464	Pilus biosynthesis protein
*fimC*	S4463	Chaperone
*fimD*	S4462	Outer membrane usher/pseudogene
*fimF*	S4458	Minor subunit
*fimG*	S4457	Minor subunit
*fimH*	S4456	Tip adhesin
Curli operon		
*csgG*	S1104	Assembly protein/pseudogene
*csgF*	S1105	Assembly protein
*csgE*	S1106	Assembly protein
*csgD*	S1107	Transcriptional regulator
***csgB***	**S1108**	**Minor subunit**
***csgA***	**S1109**	**Major subunit/pseudogene**
*csgC*	S1113	Autoagglutination assembly protein
S1114	S1114	Hypothetical (*fimA* homolog)
*ybg* operon		
*ybgD*	S0591	Fimbria-like protein (*fimA* homolog)
*ybgQ*	S0592	Outer membrane protein (*fimD* homolog)
*ybgP*	S0593	Chaperone (*fimC* homolog)
*ybgO*	S0594	Putative fimbria-protein/pseudogene
*ycb* operon		
*ycbQ*	S1003	Fimbria-like adhesin/pseudogene
*ycbS*	S1006	Outer membrane usher protein
S1007	S1007	Fimbrial protein
S1008	S1008	Fimbria-like protein
S1009	S1009	Fimbria-like protein (*fimA* homolog)
*ycbF*	S1010	Putative pilus assembly chaperone
*yeh* operon		
*yehD*	S2298	Fimbria-like protein
*yehC*	S2297	Chaperone/pseudogene
*yehB*	S2296	Outer membrane usher protein
*yehA*	S2295	Putative fimbria-protein/pseudogene
*yra* operon		
*yraH*	S3396	Fimbria-like adhesin/pseudogene
*yraI*	S3397	Chaperone (*fimC* homolog)
*yraJ*	S3398	Outer membrane usher/pseudogene
*yraK*	S3403	Fimbrial protein
S4250-S4254		
S4250	S4250	Pilus assembly chaperone (*papD_C* homolog)
S4254	S4254	Outer membrane usher/pseudogene
S3342-S3341		
S3342	S3342	Hypothetical (*fimD* homolog)
S3341	S3341	Hypothetical (pilus biogenesis initiator homolog)
*sfm* operon		
*sfmA*	S0469	Fimbria-like protein (*fimA* homolog)
*sfmC*	S0470	Chaperone
*sfmD*		Outer membrane usher/pseudogene

a Boldface entries provide information for the genes deleted in this study.

10.1128/mSphere.00751-19.2 FIG S2 Additional adherence gene cluster analysis. Analysis was performed as described in Materials and Methods as well as in the Fig. 2 legend. A 10-fold enhanced view of the trace reads are provided for genes S3342 and *sfmA* to *sfmC* to demonstrate the presence of RNA-seq reads. Download FIG S2, PDF file, 0.2 MB. Copyright © 2019 Chanin et al. 2019 Chanin et al. This content is distributed under the terms of the Creative Commons Attribution 4.0 International license.

### Mutational analyses of three S. flexneri 2457T adherence structural genes.

We next performed mutational analyses of the genes encoding major structural subunits to demonstrate functional roles in epithelial cell adherence and biofilm formation. We concentrated our analyses on genes that encode long polar fimbriae, type 1 fimbriae, and curli, based on our *in silico* analyses, the combined appearance of the structures in Fig. 1 and 2, and the known functional roles of these structures in initial biofilm formation and epithelial cell adherence in other pathogens (8, 28, 47–49). Thus, we constructed Δ*lpfA*, Δ*fimA*, and Δ*csgA* mutants. We also constructed a double Δ*csgAB* mutant due to the additional role of the *csgB* minor subunit in adherence (50).

**FIG 5:**
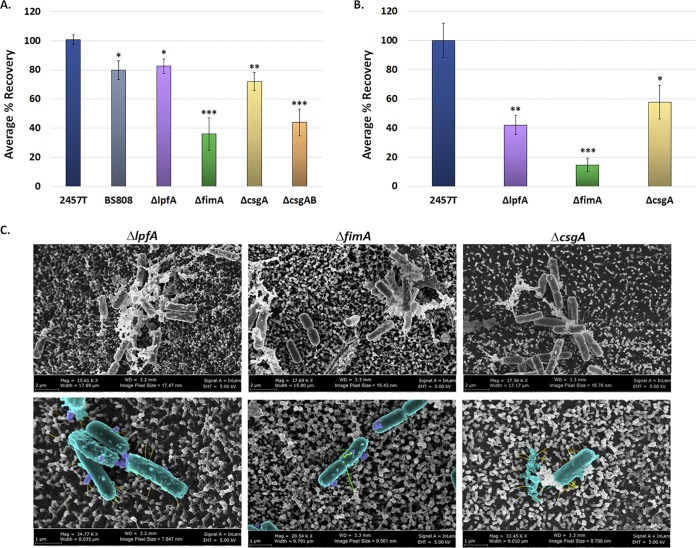
Analysis of epithelial cell adherence for each S. flexneri 2457T adherence mutant. (A) Wild-type S. flexneri strain 2457T, the control strain BS808 (Δ*ospE1* Δ*ospE2*), and the adherence mutants (the Δ*lpfA*, Δ*fimA*, Δ*csgA*, and Δ*csgAB* mutants) were grown overnight as described in the text for the biofilm assay. On the next day, bacteria were collected, washed with 1× PBS, and analyzed for adherence to HT-29 cells. Each mutant had a significant reduction in adherence relative to wild-type strain 2457T, with the most significant reductions being seen with the Δ*fimA* and Δ*csgAB* mutants. (B) To verify the data obtained with HT-29 cells, the Δ*lpfA*, Δ*fimA*, and Δ*csgA* mutants were evaluated for adherence in the HIODEM model. Like with the HT-29 cells, each mutant displayed a significant reduction in adherence relative to wild-type strain 2457T, with the Δ*fimA* mutant having the greatest reduction. For both panels A and B, the average percent recovery of adherent bacteria ± standard error relative to that of wild-type strain 2457T is plotted. The results for HT-29 cell infections represent those from three independent experiments in which each experiment had technical triplicates. The results for HIODEM infections represent those from two independent experiments in which each experiment had at least two technical replicates. *, *P* < 0.05; **, *P* < 0.01; ***, *P* < 0.001. (C) Scanning electron microscopy analysis of the Δ*lpfA*, Δ*fimA*, and Δ*csgA* mutants on the HIODEM model enabled visualization of the adherence structures for each mutant. Magnifications, ×15,600 to ×17,700 (top row) and ×28,500 to ×34,800 (bottom row). To facilitate the visualization of adherence structures, pseudocoloring was performed for the images in the bottom row, as described in the legend to Fig. 2. The Δ*lpfA* mutant lacked the thicker structures, the Δ*fimA* mutant lacked the thinner structures, and the Δ*csgA* mutant lacked the electron-dense aggregates.

Functional analyses of the mutants were performed to evaluate the role of each factor in adhesion to epithelial cells and biofilm formation. First, we hypothesized that the adherence factors expressed in the IVLCs would facilitate epithelial cell contact. This hypothesis was supported by our previous observations of induced S. flexneri 2457T adherence to HT-29 cells following biofilm dispersion under conditions that mimicked the loss of the bile salts signal during the terminal ileum-to-colon transition (31). All mutants had significant reductions in adherence relative to wild-type bacteria, with the Δ*fimA* and Δ*csgAB* mutants having the greatest reductions (Fig. 5A). The double Δ*ospE1 ospE2* mutant (strain BS808) served as an adherence mutant control, given our previous analysis of the role of OspE1 and OspE2 in bile salt-mediated adherence (30). To ensure that the mutations did not affect the overall invasive ability of each strain, invasion assays were performed using conventional methods of centrifugation to initiate host cell contact (51). All mutants retained wild-type levels of invasion following centrifugation of the bacteria onto the HT-29 cells (data not shown), which confirmed that the mutations did not affect the basic invasion phenotype of the strains. Finally, to confirm the HT-29 cell adherence data, we evaluated the Δ*lpfA*, Δ*fimA*, and Δ*csgA* mutants in the HIODEM model and found that each mutant had significantly reduced adherence relative to wild-type bacteria (Fig. 5B). EM analysis of infected samples enabled the visualization of mutants with a smoother surface and less visible structures than wild-type bacteria (Fig. 2 and 5C). Combined, the data demonstrate that the S. flexneri 2457T *lpfA*, *fimA*, and *csgAB* gene products have functional roles in adherence to colonic epithelial cells.

**FIG 6:**
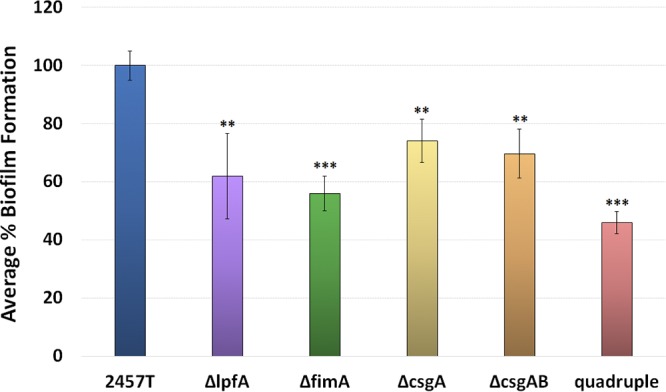
Analysis of biofilm formation for each S. flexneri 2457T adherence mutant. Wild-type S. flexneri strain 2457T and the Δ*lpfA*, Δ*fimA*, Δ*csgA*, Δ*csgAB*, and quadruple mutants were analyzed for biofilm formation at 3 h postinoculation to examine the adherence phase of biofilm formation in IVLCs. Each mutant had a significant reduction in biofilm formation. The average percent biofilm formation ± standard error relative to that for 2457T is plotted for each mutant. All data represent those from three biological independent experiments in which each experiment had at least three technical replicates. **, *P* < 0.01; ***, *P* < 0.001.

Second, given the importance of adherence in the initiation of biofilms (37, 52), we analyzed the mutants in the biofilm assay (31, 35). All mutants exhibited reduced biofilm formation at 3 h (Fig. 6), a time point used to examine the role of adherence factors in early biofilm formation (35). A quadruple mutant (the Δ*csgAB* Δ*lpfA* Δ*fimA* mutant) was constructed to confirm the results of the biofilm analyses and displayed the greatest reduction in biofilm formation relative to wild-type bacteria. Thus, we concluded that the *lpfA*, *fimA*, and *csgAB* gene products also have roles in the adhesion process for IVLC-induced biofilm formation in S. flexneri 2457T.

**FIG 7:**
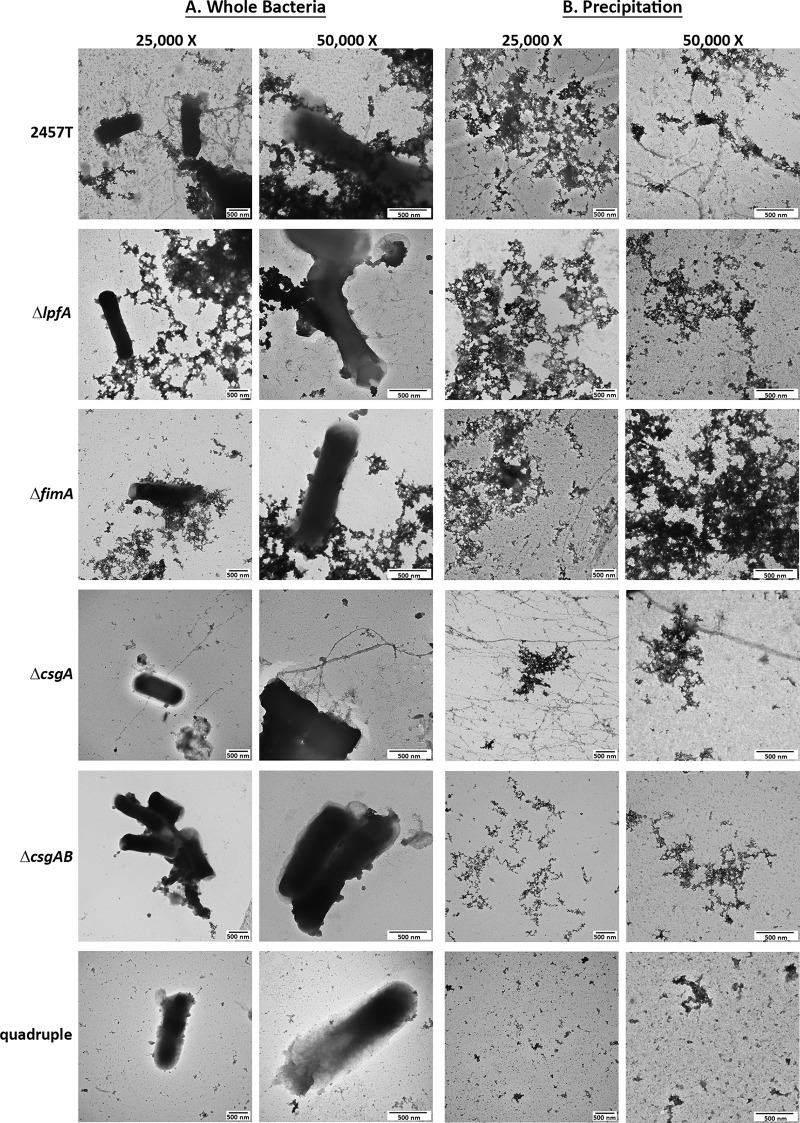
TEM analysis for each S. flexneri 2457T adherence mutant. (A) The Δ*lpfA*, Δ*fimA*, Δ*csgA*, Δ*csgAB*, and quadruple mutants were grown statically overnight in IVLCs, subsequently processed for TEM analysis, and analyzed with wild-type strain 2457T as a control. Each mutation resulted in the loss of either the thicker structures (Δ*lpfA* mutant), the thinner structures (Δ*fimA* mutant), or the electron-dense aggregates (Δ*csgA* and Δ*csgAB* mutants). The quadruple mutant did not display visible structures. Images for wild-type strain 2457T are from experiments separate and biologically independent of those used to obtain the images provided in Fig. 1. (B) To verify the results, ammonium sulfate precipitation was performed to isolate and visualize structures from wild-type S. flexneri strain 2457T and each of the five mutants. The three types of factors can be visualized in wild-type bacteria; however, only two of the three structures were present for the single mutants. Each mutation resulted in the expected loss of structure, and no structures were visualized in the quadruple mutant. The data verify that the correct structural subunit was deleted for each mutant. All images are representative of those from at least two biological independent experiments. Different fields are presented for the images with ×25,000 and ×50,000 magnifications for all images in panels A and B, in which both sets of images display the 500-nm scale bar.

Finally, the mutants were evaluated by EM for the loss of surface structures. For the single mutants, each mutation resulted in the loss of a structure, while it also facilitated visualization of two other predominant structures. No apparent structures were visualized in the quadruple mutant (Fig. 7A). Furthermore, ammonium sulfate precipitation for the isolation of adherence factors (53) was performed to verify our results (Fig. 7B). Finally, to provide additional data for the presence of a *csgA* gene product despite the disorganized appearance, the Congo red (CR) binding assay was performed, given the ability of Congo red dye to bind the amyloid structures of curli and produce a birefringence signal under polarized light (54–56). A positive birefringence signal was detected for both wild-type strain 2457T and the Δ*lpfA* mutant, which was used as a mutation control for this assay. However, the Δ*csgA* mutant produced significantly less signal (Fig. S3). In all, these analyses suggest that we identified the genes encoding the structural subunits of the putative adherence structures expressed by S. flexneri 2457T.

10.1128/mSphere.00751-19.3 FIG S3 Congo red binding assay. The assay was performed to test for the presence of the CsgA protein due to the ability of the Congo red dye to bind amyloid fibers and produce a birefringence signal under polarized light. The apple green color indicates the Congo red fluorescence that occurs when amyloid fibers are present. Wild-type 2457T produced a positive signal, while a significant reduction in the signal was detected for the Δ*csgA* mutant. The Δ*lpfA* mutant was analyzed as a mutation control, and as seen, the mutation did not affect the birefringence signal. Images are representative of those from at least two biological independent experiments. Download FIG S3, PDF file, 0.2 MB. Copyright © 2019 Chanin et al. 2019 Chanin et al. This content is distributed under the terms of the Creative Commons Attribution 4.0 International license.

### MS analysis to evaluate secretion of adherence structural proteins in IVLCs.

**FIG 8:**
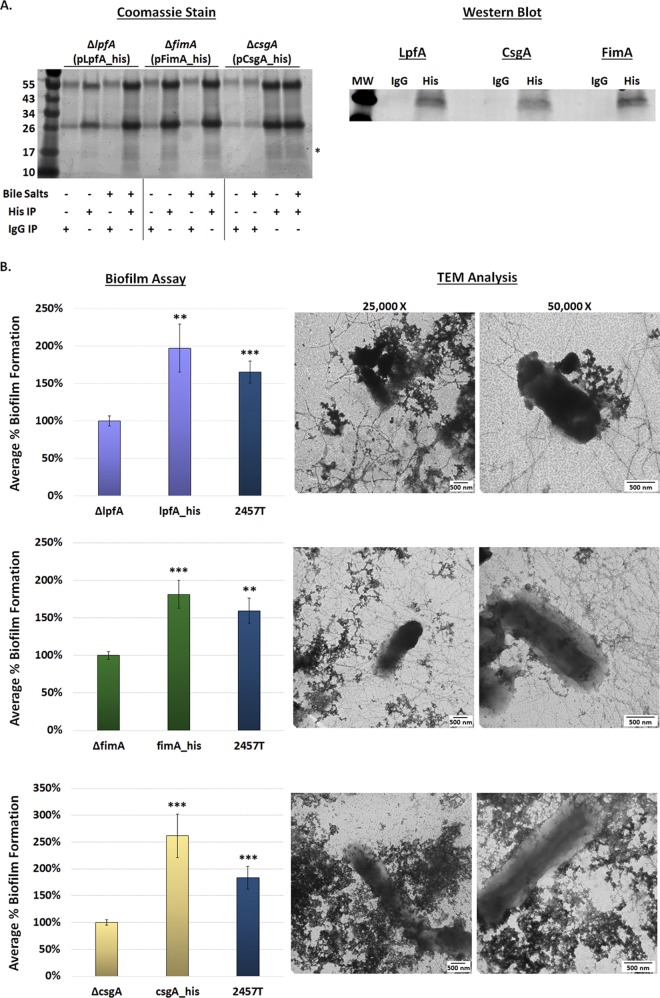
Immunoprecipitation and functional complementation of the histidine-tagged proteins. (A) To confirm the results of mass spectrometry analysis, bacteria expressing His-tagged proteins were cultured in the overnight biofilm assay and the culture supernatants were immunoprecipitated. (Left) IgG negative-control and anti-His immunoprecipitation (IP) experiments were performed in media with or without IVLCs with analysis by SDS-PAGE with Coomassie blue staining. Proteins in the 17-kDa range (*) were immunoprecipitated with the anti-His resin and in the presence of bile salts. (Right) Western blot analysis of proteins in the 17-kDa range confirmed that the His-tagged LpfA, FimA, and CsgA were secreted into the supernatant of the biofilm culture. No proteins were detected in the negative-control samples in which IgG was used in the immunoprecipitation. (B) Biofilm and TEM analyses were used to verify that the His-tagged proteins were functional and could complement the respective mutants. The complemented strains (strains complemented with *lpfA* [top], *fimA* [middle], and *csgA* [bottom]) produced significantly higher biofilms than the mutants to indicate the restoration of function, while TEM analysis enabled visualization of the overexpressed structures. The results of the biofilm assays were analyzed at 4.5 h. The data represent the average from two independent experiments, each with six technical replicates, ± standard error. **, *P* < 0.01; ***, *P* < 0.001. The level for each complemented strain averaged the same or above the level for wild-type strain 2457T (the differences were not significant). Note the differences in the *y* axis range between the analysis with the *csgA* mutant and the analyses with the *lpfA* and *fimA* mutants.

As a method to detect the presence and secretion of the LpfA, FimA, and CsgAB proteins, proteomic analyses were performed on culture supernatants from the biofilm assay. Both intact mass spectrometry (MS) analysis and peptide fingerprinting MS/MS analysis of trypsin-digested samples confirmed the presence of LpfA, FimA, CsgA, and CsgB, with each protein having high levels of sequence coverage upon the fingerprinting MS/MS analysis (Table 2), indicating that the proteins were secreted in IVLCs. Due to the complexity of the samples for MS analysis, especially those from the extracellular polymeric substance (EPS) matrix produced by the IVLC-induced biofilm (31), a higher than expected mass error was observed. Therefore, we cloned the *lpfA*, *fimA*, and *csgA* genes from S. flexneri 2457T, added a histidine tag to the genes, and transformed each respective mutant to perform immunoprecipitation and complementation analyses. As shown in Fig. 8, the tagged LpfA, FimA, and CsgA proteins were expressed in the respective mutants, secreted, and purified from IVLC-induced biofilm culture supernatants, which confirmed the MS data. Biofilm assay analyses and TEM visualization of the overexpressed structures not only verified that these tagged constructs were functional (Fig. 8) but also provided additional data to confirm the findings of the mutational and EM analyses and thus demonstrate that the *lpfA*, *fimA*, and *csgAB* genes produce functional adherence proteins in S. flexneri 2457T.

**TABLE 2:** Mass spectrometry analysis of the bile salt-induced biofilm supernatants

Protein	Intact avg mass (Da)		Mass error (ppm)	Response (total ion count)	Digested peptide coverage (%)
	Observed	Expected			
LpfA	19,644.38	19,642.80	86.8	583,652,552	100
FimA	18,348.20	18,347.28	49.8	221,292,058	82
CsgA	12,389.58	12,388.92	53.4	203,325,206	84
CsgB	15,870.81	15,869.62	74.9	369,128,136	91

## DISCUSSION

Characterization of the three structural genes in this study provides evidence that S. flexneri 2457T utilizes traditional adherence factors to initiate biofilm formation and to facilitate contact with colonic epithelial cells. Several observations influenced the investigation, including the lack of an adherence-null mutant in OspE1 and OspE2 analysis (30), the subsequent biofilm formation and induced adherence observed following IVLC exposure (31, 35), as well as the presence of the various adherence gene clusters in the S. flexneri 2457T genome. The literature on traditional *Shigella* adherence factors is contradictory. Numerous studies have suggested that the various gene clusters have been lost during evolution as a pathoadaptive response to the host. Notably, the laboratory growth methods consistently used to demonstrate fimbrial production in strains of E. coli (19, 20) were not successful for either lab strains or clinical isolates of *Shigella* (17, 18). Our analyses with control medium, in which the combination of glucose and bile salts was absent, confirmed many of these previous findings on the phenotypic level. The visualization of putative adherence structures required the addition of both glucose and bile salts to the media, signals that are present in the small intestine during host transit (31–36). Interestingly, glucose induced the transcription of the structural subunits (Fig. 4), yet adherence factors were not visible in LB medium-glucose treatment, while minimal adherence factors were visualized in the LB medium-bile salts treatment (Fig. 1). Thus, based on the data presented in Fig. 1 and 4, we hypothesize that glucose induces structural gene transcription, while bile salts serve as a secretion signal. The amount of glucose required for signaling can vary, as is evident by the different percentages of glucose in TSB and the glucose-supplemented LB medium, a finding which is consistent with our previous observations (31). Nevertheless, this work highlights the importance of using physiological conditions to study bacterial pathogenesis, especially for human-adapted pathogens like *Shigella*.

The combined RNA-seq and RT-PCR analyses of the adherence gene clusters demonstrate that some of the gene annotations are accurate, while other annotations require refinement. For example, the *csgG* gene is annotated as a pseudogene due to a point mutation that creates an in-frame stop codon. The RT-PCR analysis confirmed this annotation, since a partial *csgG* product was detected prior to the stop codon; however, no product was detected with a reverse primer that annealed downstream of this mutation. As another example, there was significant transcription of the *ycbQ* gene, despite the truncated pseudogene annotation. Finally, while the full *ybgO* gene could not be amplified under the conditions examined, inspection of the primary genomic sequence (GenBank accession number AE014073.1) combined with the RNA-seq read mapping indicates that two separate open reading frames or small RNAs may be transcribed in this region. The effects of transcription of these partial gene fragments on S. flexneri 2457T gene regulation or adherence factor expression will require additional analyses.

The mutational and complementation analyses demonstrated functional adherence roles for the products of the *lpfA*, *fimA*, and *csgAB* structural genes. While additional direct evidence of each structure is required and such comprehensive studies are under way, the EM, mutational, and complementation analyses provided significant support for the appearance and function of each structure. Long polar fimbriae have been shown to be important for pathogenic E. coli and *Salmonella* interactions with M cells during intestinal colonization and can facilitate biofilm formation in pathogenic E. coli strains. The *lpfA* genes have also been demonstrated to be induced by bile salts (13, 57–61). As seen in Fig. 2, thicker structures are bound to the surface of cells lacking microvilli, which is a hallmark of M cells (62). Additionally, the Δ*lpfA* mutation had a greater effect on adherence in the HIODEM model, in which M cells are present (38–41), than on adherence to HT-29 cells (Fig. 5). For type 1 fimbriae, previous studies support our observations of both *fimA* gene transcription and soluble FimA expression. First, clinical isolates of *Shigella* produced fimbria-like adhesins after periods of prolonged static growth; however, the genes encoding the factors were not identified (28). Second, another RNA-seq study detected significant induction of the type 1 *fim* operon in a Δ*icgR* mutant of S. flexneri 2457T during the intracellular phase of the *Shigella* lifestyle (63). Finally, soluble S. flexneri FimA protects mitochondrial integrity and epithelial cell survival during infection (64). It is worth noting that the predicted type 1 fimbria-like structures visualized from the biofilm assays (Fig. 1 and 7) appeared to be thinner than the fimbria-like structures visualized during infection (Fig. 2 and 5). We hypothesize that the structures may appear atypical relative to observed E. coli structures, especially since a truncated or substituted FimD (see below) could affect assembly. While the Δ*fimA* mutant analyses resulted in fewer visualized fimbria-like structures (Fig. 5 and 7), we currently cannot rule out the possibility of the contribution of or compensation by the additional S. flexneri 2457T *fimA* homologs or other genes (Table 1), particularly under bile salt conditions that induce such a strong biofilm response (31, 65). For example, it was recently demonstrated that the IcsA autotransporter protein facilitates S. flexneri cell-cell interactions in bile salt-induced biofilms (66). Nevertheless, as was the case for strains complemented with *lpfA* and *csgA*, analysis of the histidine-tagged *fimA*-complemented strain provided further data on the appearance of the structures while verifying their function (Fig. 8). Thus, there is strong evidence that the type 1-like fimbriae visualized in our analyses are due to expression from the *fimA* structural gene.

The proteins generated by the *csgAB* genes in S. flexneri 2457T appear to be disorganized and to have a limited organization compared to the conventional curli fiber structures detected in other pathogens (47, 54). This lack of complete assembly could be due to a truncated CsgA protein or due to the incomplete production of CsgG, the outer membrane lipoprotein involved in the stability of the curlin proteins during assembly (22, 47, 67). Furthermore, a truncated CsgG may prevent appropriate interaction with CsgF, thereby affecting curli assembly (47, 68). Our analyses indicate that a soluble portion of CsgA is produced in S. flexneri 2457T and that it is sufficient to provide function in adherence, particularly in the establishment of the IVLC-induced biofilm. This soluble portion of the CsgA protein is likely facilitated by a functional CsgB minor subunit protein, given the further reduction in phenotypes of the double Δ*csgAB* mutant, the visualization of electron-dense aggregates in the Δ*csgA* mutant (Fig. 5 and 7), and the demonstration that CsgB has a role in adherence (50). Interestingly, our EM images suggest that *csgAB* products may exploit other adherence structures as a scaffold for a more appropriate organization (e.g., see the rough, complex structures marked by asterisks in Fig. 1B). Moreover, the additional electron-dense material visualized in the mutants, particularly with the Δ*csgAB* mutant and the quadruple mutants in Fig. 7, is likely from the cellulose component of the EPS matrix, which is also controlled by the transcriptional regulator CsgD (69). Treatment of the S. flexneri 2457T IVLC-induced biofilm with cellulase, which hydrolyzes β-1,4 glycosidic linkages (70), resulted in a significant reduction in the IVLC-induced biofilm (see Fig. S4 in the supplemental material). Further characterization of the electron-dense material is required, but the combined EM analyses (Fig. 5, 7, and 8) demonstrate that much of this material is due to the presence of the *csgA* and *csgB* genes. Thus, regardless of the appearance, our data demonstrate that the *csgAB* products are produced in S. flexneri 2457T and have functional roles in adherence.

10.1128/mSphere.00751-19.4 FIG S4 Treatment of the IVLC-induced biofilm with cellulase. The biofilm formation analysis was performed with or without cellulase to analyze the contribution of cellulose. A significant reduction in biofilm formation was detected in the presence of cellulase. All data represent those for three average OD_540_ readings from three biological independent experiments in which each experiment had technical triplicates. ***, *P* < 0.001. Download FIG S4, PDF file, 0.04 MB. Copyright © 2019 Chanin et al. 2019 Chanin et al. This content is distributed under the terms of the Creative Commons Attribution 4.0 International license.

**FIG 9:**
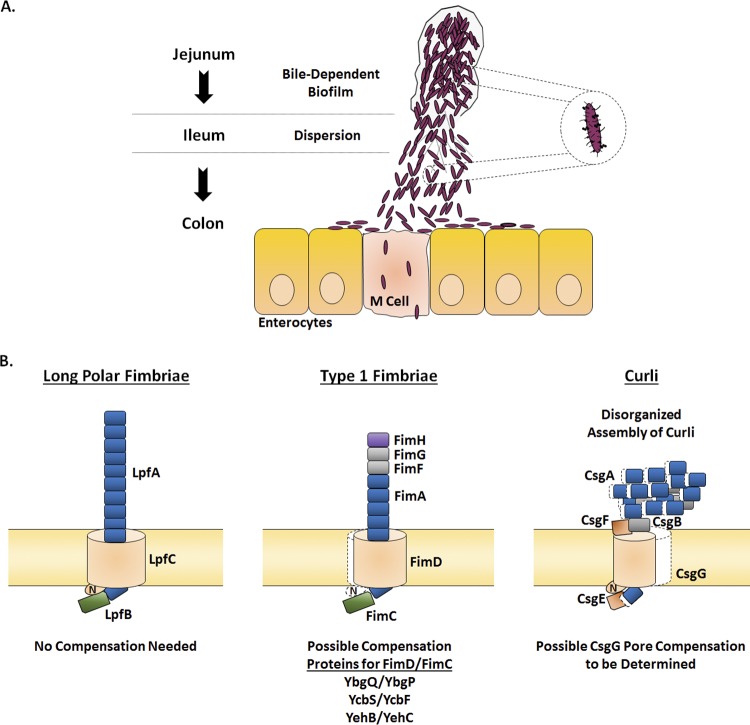
Hypothesized model of Shigella flexneri adherence factor expression during infection. (A) Based on our combined analyses performed previously (31, 35) and here, we propose the following infection model. *Shigella* enters the small intestine and resists the initial bile salts exposure that occurs in the duodenum. As food is digested in the duodenum and jejunum, free glucose becomes available. The combined bile salts and glucose signals result in adherence factor expression and biofilm formation. Following transit through the majority of the small intestine, biofilm dispersion is triggered by loss of the bile salts signal in the terminal ileum and colon. Subsequently, *Shigella* adheres to enterocytes and M cells to initiate the infection process. The figure was adapted from our previous publication (31). (B) The hypothesized protein organization for each of the three adherence factors based on the analyses performed in this study is provided. First, the major components for the long polar fimbriae are likely intact and functional. Second, we hypothesize that the major and minor structural subunits for type 1 fimbriae are intact to form a full structure. Due to the insertion sequence in *fimD*, we predict either a truncated protein in which the N terminus is missing (highlighted by the white shadow relative to E. coli) or compensation by one of the other FimD homologues listed to enable secretion of the structural subunits. Depending on the truncation, FimC may still be able to interact with FimD or the homologues to serve the appropriate chaperone function. Otherwise, the corresponding FimC homologue would be utilized. Third, due to the N-terminal CsgA truncation and/or the C-terminal CsgG truncation, we hypothesize secretion but not the complete assembly of CsgA and CsgB for curli. Curli assembly may also be affected by a possible lack of CsgF interaction with a truncated CsgG. Colors for the proteins correspond to the color scheme described in the legend to Fig. 3.

The pseudogene annotations, particularly for the genes encoding the pores or chaperone-usher components required for assembly of the major structural subunits, warrant future investigations into determining how S. flexneri 2457T assembles adherence structures (Fig. 9). If *fimD* is nonfunctional, we hypothesize that homologous genes located in other genomic locations may compensate for a pseudogene in an operon, if needed. For example, the *ybgQ*, *ycbS*, or *yehB* ushers and accompanying chaperone genes may compensate for the truncated expression of *fimD* in the *fim* operon to enable FimA secretion and assembly. This hypothesis is supported by the demonstration of fimbrial promiscuity in biogenesis in E. coli, in which heterologous structural subunits or secretion systems from different operons are utilized to generate and assemble intact structures (71, 72). Fimbrial promiscuity has also been suggested for Proteus mirabilis, since soluble Fim14A was detected by MS in the extracellular environment, despite an incomplete operon in which the chaperone is absent and the usher is annotated as a pseudogene. Proteus mirabilis encodes 17 chaperone-usher fimbrial operons, and therefore, compensation by one of the other operons is hypothesized to enable Fim14A secretion (73). Thus, functional products are likely produced by the other S. flexneri 2457T operons, especially for the ushers, given the transcriptomic analyses performed and the identification of at least three *fimD* homologs throughout the genome, as denoted by the color coding in Fig. 3 and Fig. S2.

In conclusion, we have demonstrated that at least three S. flexneri 2457T adherence structural genes generate functional products for IVLC-induced biofilm formation and adherence to colonic epithelial cells, despite the presence of any mutations that would normally inhibit expression. Future investigations, including in-depth analyses defining the mechanism of adherence factor production and secretion in IVLCs, the effects of an anerobic environment as the bacteria transition to the colon, and studies with other *Shigella* species, will enhance our understanding of the evolution of this pathogen. Analysis of two clinical S. flexneri isolates thus far demonstrated conserved phenotypes (Fig. S5). We hypothesize that the pathoadaptive changes that *Shigella* sustained were not the loss of adhesion expression but, rather, a precise control of gene expression to enable the production of adhesins only when necessary and in instances that are most beneficial to the pathogen. We agree that constitutive expression of these adherence factors would possibly interfere with the pathogenic lifestyle of *Shigella* and impair critical immune evasion tactics. A similar regulation of adhesion genes has been described for other bacterial pathogens, such as enterotoxigenic, enterohemorrhagic, and uropathogenic E. coli strains (74–78). Clearly, human-adapted pathogens have efficiently evolved to regulate virulence gene expression for efficient colonization and infection tactics in the human host. Our work provides an example of this concept and highlights the importance of utilizing IVLCs to study bacterial pathogens. Finally, this work has profound effects on the development of therapeutics against *Shigella*. The adherence factors provide innovative targets and promise for novel therapies and new strategies to ultimately control and prevent *Shigella* infection.

10.1128/mSphere.00751-19.5 FIG S5 Analysis of clinical isolates of Shigella flexneri. Both TEM (top) and biofilm formation (bottom) analyses were performed with clinical isolates of S. flexneri serotype 3a (strain AF11) and S. flexneri serotype 2a (strain AF16) to confirm the results of the study. Magnification for TEM, ×50,000 (on the left for strain AF11) and ×25,000 (on the right for strain AF16). For the biofilm assay, the average OD_540_ for the strains grown in either medium (light blue bars) or medium supplemented with IVLCs (dark blue bars) is plotted. All data represent those from three biological independent experiments in which each experiment had technical triplicates. ***, *P* < 0.001 for TSB versus TSB-bile salts for each strain. Download FIG S5, PDF file, 0.5 MB. Copyright © 2019 Chanin et al. 2019 Chanin et al. This content is distributed under the terms of the Creative Commons Attribution 4.0 International license.

## MATERIALS AND METHODS

### Ethics statement.

Human sample collection was approved by Institutional Review Board (IRB) protocol 2015P001908 of the Massachusetts General Hospital, Boston, MA. Donor tissue was obtained from consenting patients undergoing medically required surgical resections, as determined by a licensed physician. All subjects provided written informed consent.

### Bacterial strains and growth conditions.

The bacterial strains and plasmids used in this study are presented in Table 3. Bacteria were routinely cultured at 37°C in either Luria broth (LB; Lennox) or tryptic soy broth (TSB; which contains an additional 2.5 g/liter glucose relative to LB) with aeration or in tissue culture-treated plates to represent static growth conditions (31, 35). Plating for determination of the number of CFU was performed using tryptic soy broth plates with 1.5% agar and 0.025% Congo red (CR; catalog number C6277; Sigma-Aldrich). Bile salts (catalog number B8756; Sigma-Aldrich) were used at a concentration of 0.4% (wt/vol). All media were filter sterilized with a 0.22-μm-pore-size filter following the addition of bile salts and/or glucose. Chloramphenicol was used at 5 μg/ml, kanamycin was used at 50 μg/ml, and ampicillin was used at 100 μg/ml, where indicated.

**TABLE 3:** Strains and plasmids used in this study

Strain or plasmid	Description	Source or reference
*Shigella* strains		
2457T	S. flexneri serotype 2a	92
BS808	2457T *ospE1*::*aphA-3 ospE2*::*cat*	30
BS766	2457T transformed with pKM208	89
Δ*lpfA*	2457T *lpfA*::*cat*, chloramphenicol resistance	This study
Δ*fimA*	2457T *fimA*::*aph-3*, kanamycin resistance	This study
Δ*csgA*	2457T *csgA*::*cat*, chloramphenicol resistance	This study
Δ*csgAB*	2457T *csgB csgA*::*aph-3*, kanamycin resistance	This study
Δ*csgAB* Δ*lpfA* Δ*fimA*	2457T Δ*csgAB lpfA*::*cat fimA*::*aph-3*, chloramphenicol and kanamycin resistance	This study
Δ*lpfA*(pLpfA_his)	2457T *lpfA*::*cat* transformed with pLpfA_his	This study
Δ*fimA*(pFimA_his)	2457T *fimA*::*aph-3* transformed with pFimA_his	This study
Δ*csgA*(pCsgA_his)	2457T *csgA*::*cat* transformed with pCsgA_his	This study
AF11	S. flexneri serotype 3a (recent clinical isolate)	AFRIMS^*a*^
AF16	S. flexneri serotype 2a (recent clinical isolate)	AFRIMS
Plasmids		
pKD3	*oriR6K bla cat*	93
pKD4	*oriR6K bla aph-3*	93
pKM208	Temperature-sensitive *red*-, *gam*-, and *lacI*- expressing plasmid driven by *P_tac_* promoter, *bla*	93
pCP20	FLP^+^ λ *c*I857^+^ λ ρ_R_ Rep^Ts^ *bla cat*	90
pGEMT	PCR cloning vector, *bla*, high copy number	Promega
pLpfA_his	pGEMT::*lpfA lpfA* with C-terminal His tag	This study
pFimA_his	pGEMT::*fimA fimA* with C-terminal His tag	This study
pCsgA_his	pGEMT::*csgA csgA* with C-terminal His tag	This study

a AFRIMS, Department of Enteric Diseases, Armed Forces Research Institute of Medical Sciences, Bangkok, Thailand.

### Biofilm assays.

The biofilm assay was performed as previously described (31, 35). Single colonies of each bacterial strain were inoculated into IVLC medium in a single well of a 96-well plate. The plates were incubated at 37°C without shaking. At the time points indicated below, the wells were gently washed twice with 1× phosphate-buffered saline (PBS) and either fixed for electron microscopy (see below) or stained with 0.5% crystal violet for 5 min. Afterwards, the wells were gently washed five times with sterile distilled H_2_O and then set to air dry. Biofilm formation was quantified by adding 95% ethanol to the wells to solubilize the crystal violet stain. After 30 min of incubation at room temperature on an orbital shaker at 70 rpm, the absorbance reading at an optical density of 540 nm (OD_540_) was measured with a plate reader (79). Absorbance readings at OD_600_ were taken to ensure that there were no significant differences in growth prior to the washing steps. For experiments in which cellulase (catalog number C1184; Sigma-Aldrich) was used, 60 units/ml of enzyme was added to the wells at the start of the biofilm assay. For complementation analysis, the assays were performed at 4.5 h to enable appropriate expression of the genes from the pGEMT plasmid. Cellulase and complementation biofilms were subsequently processed as described above. Statistical significance was determined by comparing the findings for wild-type S. flexneri 2457T to those for each mutant using Student's *t* test, and a *P* value of ≤0.05 was considered significant.

### Adherence assays.

The isolation and preparation of human intestinal epithelial cells were performed as previously described (38–41, 80, 81). The excess healthy margins of the ascending colon, as verified by a pathologist, were used to obtain the intestinal crypts. The tissue was washed once in cold 1× PBS (Thermo Fisher Scientific, MA), and then tissue strips were cut and placed into a dissociation buffer consisting of 1× PBS, penicillin-streptomycin (pen-strep; Thermo Fisher Scientific), 1 mM dithiothreitol (DTT; Sigma-Aldrich, MO), and 0.5 mM EDTA (Sigma-Aldrich). Intestinal strips were incubated at 4°C for 30 min and then vigorously shaken to promote epithelium dissociation from the basal membrane. This procedure was repeated five times to collect multiple fractions. Subsequently, the fractions containing the intestinal crypts were further processed and plated in Matrigel matrix (Corning, NY) as previously described (38, 80). Intestinal crypt-derived organoids were incubated at 37°C with 5% CO_2_ in medium that consisted of a 1:1 ratio of stem cell medium and L-WRN (ATCC CRL-3276)-derived conditioned medium, in which both medium types were prepared as previously described (38, 82). The culture medium was replenished every other day, and the organoids were passaged every 7 to 9 days using standard trypsin-based techniques. Approximately 2.0 × 10^6^ cells/ml were replated in Matrigel matrix to ensure robust propagation of the organoids (38).

Organoid-derived cell monolayers were generated as previously described (38–41). Single-cell suspensions derived from the organoids were plated on polyethylene terephthalate (PET) membrane transwell inserts with a 0.4-μm pore size (Corning) at 1.0 × 10^6^ cells/ml and incubated in the 1:1 stem cell medium–L-WRN medium at 37°C with 5% CO_2_. The culture medium was changed every other day until the cultures reached confluence, as determined by transepithelial electrical resistance (TEER) monitoring and microscopic observation. At 48 h prior to each experiment, the media in the apical chamber were replaced with complete Dulbecco modified Eagle medium (DMEM)–Ham’s F-12 medium plus 5 μM γ-secretase inhibitor IX (DAPT; Calbiochem), while the basolateral media were replenished with the 1:1 stem cell medium–L-WRN medium with 10 μM Y-27632 (Calbiochem) and 100 to 500 ng/ml of the receptor activator of the NF-κB ligand (RANKL; Peprotech). This process was utilized to promote cell differentiation (38, 40), especially for M cells (83). On the day of each experiment, the monolayers were washed with 1× PBS, both the apical and basolateral media were replaced with DMEM without phenol red, and the monolayers were incubated for at least 2 h before the initiation of the experiment. S. flexneri 2457T or the various mutants were subcultured in TSB-bile salts and then were washed in 1× PBS, resuspended in DMEM without phenol red, applied to the apical surface of the monolayers without centrifugation, and incubated for 3 h as previously described for polarized T84 cells (30). Afterwards, infected cells were processed for adherence quantification (30) or fixed for electron microscopy (see below). The average percent recovery was calculated from two independent experiments, each of which was performed with at least two technical duplicates, as (recovered bacterial titer/infecting titer) × 100% and was plotted relative to the results for wild-type strain 2457T. Statistical significance was determined by comparing the result for wild-type S. flexneri 2457T to that for each mutant using Student's *t* test, and a *P* value of ≤0.05 was considered significant.

The HT-29 cell adherence assay was performed as previously described (31). HT-29 cells (ATCC HTB-38) were seeded in DMEM to establish a semiconfluent monolayer of approximately 75%. For bacterial cultures, single colonies of S. flexneri 2457T or the various mutants were inoculated into media with or without IVLCs in tissue culture plates and grown statically at 37°C. Following overnight growth, the bacteria were collected, washed with 1× PBS, standardized to an OD_600_ of 0.35, resuspended in DMEM, and applied to the HT-29 cell monolayers without centrifugation. The cells were incubated at 37°C with 5% CO_2_ for 3 h. Afterwards, the monolayers were washed five times with 1× PBS and lysed with 1% Triton X-100. Serial dilutions were made to determine the number of cell-associated bacteria. The average percent recovery was calculated from three independent experiments as (recovered bacterial titer/infecting titer) × 100% and was plotted relative to the results for wild-type 2457T. Statistical significance was determined by comparing the result for wild-type S. flexneri 2457T to the result for each mutant using Student's *t* test, and a *P* value of ≤0.05 was considered significant.

### Electron microscopy analyses.

For the biofilm culture analysis, single colonies of S. flexneri 2457T or the various mutants were added to tissue culture-treated plates containing medium with or without IVLCs. The cultures were grown statically overnight at 37°C. On the following day, samples were collected and prepared for transmission electron microscopy (TEM) imaging by fixing in 2.5% glutaraldehyde and staining with uranyl acetate (84). Samples were mounted on Formvar/carbon 100-mesh grids (Electron Microscopy Services) and imaged with a JEOL transmission electron microscope. For scanning electron microscopy (SEM) analysis of the HIODEM adherence assay, samples were fixed in 0.5× Karnovsky fixative and subsequently stored in 1× PBS. All sample processing occurred at the Massachusetts Eye and Ear Infirmary core facility. All SEM imaging was performed at the Harvard University Center for Nanoscale Systems (CNS) using a FESEM Supra55VP microscope. The SEM images were pseudocolored according to protocols listed at http://www.nuance.northwestern.edu/docs/epic-pdf/Basic_Photoshop_for_Electron_Microscopy_06-2015.pdf.

For TEM analysis of isolated adherence structures, the wild-type and mutant strains were cultured statically in IVLC medium and an ammonium sulfate precipitation was performed (53). Briefly, samples were collected and pelleted by centrifugation at 4,000 rpm for 10 min. The bacterial pellet was resuspended in 1× PBS, and the mixture was heated at 65°C for 30 min and subsequently centrifuged at 4,000 rpm for 10 min. The supernatants were transferred to a new tube and precipitated by mixing the samples with 40% ammonium sulfate on an end-over-end mixer for 10 min at room temperature. Afterwards, the samples were dialyzed in 1× PBS using 3.5-molecular-weight-cutoff dialysis cassettes for 1 h at room temperature on a rotating shaker at 50 rpm. The 1× PBS was then changed and the cassettes were transferred to 4°C for overnight dialysis. The dialyzed fraction was collected and stored at −20°C. A fraction of each sample was fixed and processed for TEM analysis.

### RNA isolation.

RNA was isolated from the bacterial cultures as previously described (85) with Qiagen RNeasy kits. DNA was digested with Turbo DNase (Invitrogen), and the concentrations of total RNA were determined using a NanoDrop ND-1000 spectrophotometer. The cDNA was synthesized from total RNA using a SuperScript III first-strand synthesis kit (Invitrogen) or a RevertAid cDNA first-strand synthesis kit (Thermo Fisher Scientific) according to the manufacturers’ protocols. All RNA was first confirmed to be free of DNA contamination by performing separate cDNA synthesis reactions with and without reverse transcriptase in the reaction mixture, followed by PCR amplification of the housekeeping gene *rpoA* as described previously (31).

### RNA-seq analysis.

The data for the RNA sequencing (RNA-seq) trace reads were obtained from our previous study (31). Duplicate cultures were grown either by static or shaking growth in TSB or TSB supplemented with 0.4% bile salts as previously described (31). The RNA-seq trace read data were generated using Integrative Genomics Viewer (IGV) software (version 2.3.67) (86, 87). Each RNA-seq data set was loaded into IGV software, and the traces were normalized to the trace for the S. flexneri 2457T *rpoA* gene on the autoscale setting. The zoomed-in traces for two genes provided in Fig. S1 in the supplemental material represents a 10-fold magnification in the scale setting. The genes of interest were searched for using the publicly available S. flexneri 2457T genome (GenBank accession number AE014073.1) and S. flexneri serotype 2a strain 301 virulence plasmid annotations (GenBank accession number AF386526.1).

### RT-PCR analysis.

For nonquantitative reverse transcription-PCR (RT-PCR) analysis, cDNA was synthesized from total RNA isolated from broth cultures using the RevertAid cDNA first-strand synthesis kit (Thermo Fisher Scientific) according to the manufacturer’s protocol. All RNA was first confirmed to be free of DNA contamination as described above. The various PCRs were performed using the 2× Taq-Pro complete PCR mix (Denville Scientific). All primer sets were validated and tested for proper DNA amplification prior to the experiment (data not shown). The annealing temperatures were adjusted accordingly for each primer set, and the extension time was adjusted for the size of each product. The products of the reactions were visualized by gel electrophoresis on 1% agarose gels stained with ethidium bromide on a Syngene GelDoc system. The molecular weight markers used in the analysis included GeneRuler, 1 kb Plus, and 100 bp Plus (Thermo Fisher Scientific). For quantitative RT-PCR (qRT-PCR) analysis, biologically independent RNA samples were isolated, and it was ensured that they were DNA free as described above. Analysis by qRT-PCR was performed as previously described (85), and all data were normalized to the levels of *rpoA* and analyzed using the comparative cycle threshold (Δ*C_T_*) method (88). The expression levels of the target genes under the various conditions were compared using the relative quantification method (88). Real-time data are expressed as the relative changes in expression levels compared with the levels in the media without glucose and/or bile salts. Statistical significance was determined using Student's *t* test to compare the expression of each gene in control versus treatment media, and a *P* value of ≤0.05 was considered significant. Due to the significant number of primers used in this analysis, primer sequences are not presented here but are available upon request.

### Mutant construction.

The single gene deletion mutants used in this study were constructed using the bacteriophage λ Red linear recombination method as previously described (89). Briefly, PCR was used to amplify a chloramphenicol resistance cassette gene (*cat*) from pKD3 or the kanamycin resistance gene cassette (*aph-3*) from pKD4 (Table 3) with 5′ and 3′ overhangs identical to the 5′ and 3′ regions of each gene of interest. Antibiotic-resistant recombinants were identified and selected on chloramphenicol or kanamycin plates and subsequently screened via PCR using confirmation primers that annealed to unique regions up- and downstream of each gene to detect the size difference due to the insertion of the antibiotic resistance cassette. The sequences of the primers used for mutant construction and confirmation are also available upon request. For the quadruple mutant, the kanamycin resistance cassette was removed from the Δ*csgAB* mutant by transforming the strain with pCP20 (Table 3) and incubating positive transformants at 42°C as previously described (90). The strain was retransformed with pKM208, and a Δ*lpfA* deletion was constructed by inserting the chloramphenicol resistance cassette with the bacteriophage λ Red linear recombination method. Finally, this Δ*csgAB* Δ*lpfA* strain was used as the recipient strain in a bacteriophage P1L4 transduction in which the Δ*fimA* mutant was used as the donor strain and kanamycin was used to select for positive transductants. The resulting strain has a quadruple mutation in the *lpfA*, *fimA*, *csgA*, and *csgB* structural genes harboring both chloramphenicol and kanamycin resistance cassettes (Table 3). At each step of the construction, the same confirmation primers used for the single gene deletions were again used to confirm the removal or addition of the antibiotic resistance cassettes as previously described (89).

### Plasmid construction.

The plasmids harboring the sequences encoding histidine-tagged LpfA, FimA, and CsgA were constructed as previously described (30). Briefly, each gene and respective native promoter region were amplified by PCR with high-fidelity *Taq* polymerase (Invitrogen) from wild-type strain 2457T. For FimA, a 6× His tag was added to the C terminus followed by a stop codon. For LpfA and CsgA, a glycine linker sequence was added upstream of the 6× His tag. The PCR products were ligated into pGEMT, and the plasmids were subsequently transformed into the appropriate adherence mutant. Selection for positive transformants occurred on tryptic soy broth plates containing 1.5% agar, 0.025% Congo red, and 100 μg/ml ampicillin. Sequencing was performed to ensure that no mutations were introduced during the cloning process. All primers used for the plasmid constructions and sequencing verification will also be made available upon request.

### Congo red binding assay for curli detection.

Samples for the Congo red binding assay were collected by gentle scraping of samples from the biofilm, and the samples were processed for ammonium sulfate precipitation as detailed above and placed on a clean, dry glass slide. The specimens were air dried, subsequently stained with alkaline Congo red solution (catalog number HT603; Sigma-Aldrich), and incubated at room temperature for approximately 10 min. The smears were rinsed in water until excess stain was drained, and the slides were observed under polarized light for apple green birefringence (55, 56). Samples were imaged with a Nikon Ci-E microscope with an attached camera.

### Mass spectrometry analysis.

Shigella flexneri 2457T was cultured in IVLC media as described above for the biofilm assay. Following overnight incubation, culture supernatants were collected and concentrated by trichloroacetic acid (TCA) precipitation. The protein pellet was stored at −20°C until analyzed. For mass spectrometry (MS) analysis, first, intact mass analysis was performed by reconstituting the lyophilized sample in 0.1% trifluoroacetic acid. Ultra-high-pressure liquid chromatography–quantitative time of flight (Q-TOF) MS was performed to detect the masses of intact molecules present in the mixture. The samples were analyzed using reversed-phase liquid chromatography (RPLC) and a Xevo G2-S Q-TOF system (Waters Corp., Milford, MA). Liquid chromatography was performed at 0.200 ml/min using an H-Class Acquity UPLC system (Waters Corp., Milford, MA) on a BEH300-C_4_ column (2.1 mm by 150 mm; pore size, 1.7 μm; Waters Corp., Milford, MA). Buffer A consisted of 0.1% (vol/vol) formic acid in UPLC-grade water, and buffer B consisted of 0.1% (vol/vol) formic acid in 100% UPLC-grade acetonitrile. In all analyses, a gradient separation was performed as follows: 0 min 90% buffer A, 5 min 90% buffer A, 80 min 10% buffer A, 90 min 10% buffer A, 91 min 90% buffer A, and 100 min 90% buffer A. After RPLC, samples were introduced via an electrospray ion source in-line with the Xevo G2-S Q-TOF system. External calibration of the *m/z* scale was performed using sodium cesium iodide. The Q-TOF parameters were run in sensitivity mode with *m/z* scanning from 400 to 4,000, a 3.00-kV capillary voltage, a 40-V cone voltage, a 150°C source temperature, a 350°C desolvation temperature, and a desolvation gas flow rate of 800 liters/h. MS data were collected at a scan speed of 1.0 s. Liquid chromatography (LC) solvents were UPLC grade, and all other chemicals were of analytical grade. Intact masses were calculated using the Waters UNIFI software package and deconvolved using the MaxEnt algorithm.

For peptide analysis, samples were digested with trypsin at 37°C for 1.5 h, and the resulting peptides were subsequently extracted for analysis. UPLC–Q-TOF MS/MS was performed to detect the masses of the digested peptides and the respective fragments. Samples were analyzed using RPLC as described above on a BEH300-C_18_ column (2.1 mm by 150 mm; pore size, 1.7 μm; Waters Corp., Milford, MA) using buffer A and buffer B with the same compositions described above. In all analyses, a gradient separation was performed as follows: 0 min 95% buffer A, 2 min 95% buffer A, 55 min 40% buffer A, 64 min 10% buffer A, 74 min 10% buffer A, 75 min 95% buffer A, and 90 min 95% buffer A. After RPLC, samples were introduced via an electrospray ion source in-line with the Xevo G2-S Q-TOF system. External calibration of the *m/z* scale was performed using sodium cesium iodide. The Q-TOF parameters were run in resolution mode with *m/z* scanning from 50 to 2,000, a 3.00-kV capillary voltage, a 30-V cone voltage, a 130°C source temperature, a 250°C desolvation temperature, and a desolvation gas flow rate of 800 liters/h. MS/MS data were collected at a scan speed of 0.1 s. LC solvents were UPLC grade, and all other chemicals were of analytical grade. Peptide fingerprinting was completed by use of the Waters UNIFI software package. Parameters were set to restrict matches only to those peptide fragments where the primary ion exhibited a >+1 charge and at least 1 daughter ion was detected, confirming the presence of each particular peptide. Any peptide maps with less than 10% coverage were excluded from the analysis.

### Immunoprecipitation analysis.

Each strain harboring the His-tagged constructs (Table 3) was grown in static overnight biofilm cultures as described above. For plasmid maintenance, ampicillin was added. Culture supernatants were subsequently collected, filtered sterilized, and TCA precipitated. The total protein pellets were resuspended in 1 ml NP-40 with protease inhibitor cocktail (Roche Diagnostics GmbH). Samples were precleared using protein A/G plus agarose beads (Pierce), followed by immunoprecipitation with a mouse anti-His affinity resin (GenScript) or a negative-control mouse IgG antibody (Santa Cruz). Samples were incubated overnight at 4°C with rotation. On the following day, protein A/G plus agarose beads were added to the negative-control IgG samples, and the mixture was incubated for 1 h at 4°C with rotation. Afterwards, the beads or resin samples were pelleted, washed six times, and boiled in acidified Laemmli lysis buffer as previously described for adherence proteins (91). After boiling, the samples were neutralized with basic Laemmli lysis buffer. Samples were run on a 4 to 20% SDS-PAGE gel (Bio-Rad), and Western blot analysis was performed as previously described (30) using a primary anti-His antibody (Qiagen) and a secondary Alexa Fluor 700 goat anti-mouse immunoglobulin antibody. The Western blots were scanned using an Odyssey infrared detection system (Li-Cor).
