# Supplementary material for: Shigella flexneri Adherence Factor Expression in In Vivo-Like Conditions
Source: mSphere. 2019 Nov 13;4(6):e00751-19. doi: 10.1128/mSphere.00751-19 (PMC6854044; doi:10.1128/mSphere.00751-19)

Supplemental Figure S1

A. Additional images of *S. flexneri* 2457T

80,000 X

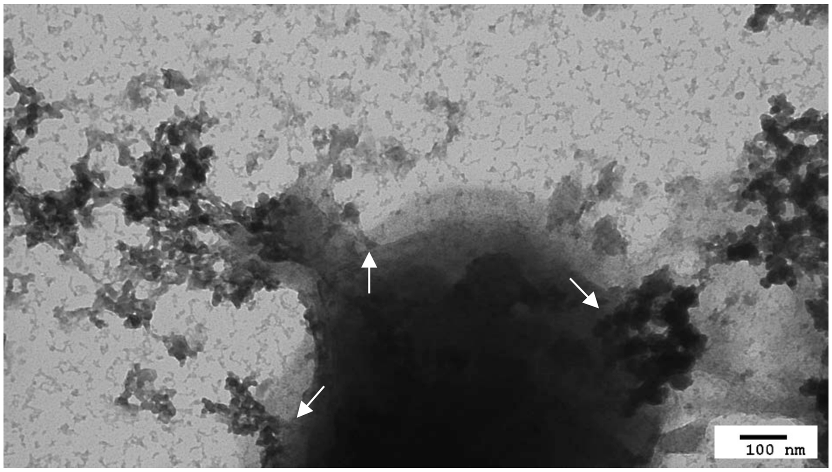

50,000 X

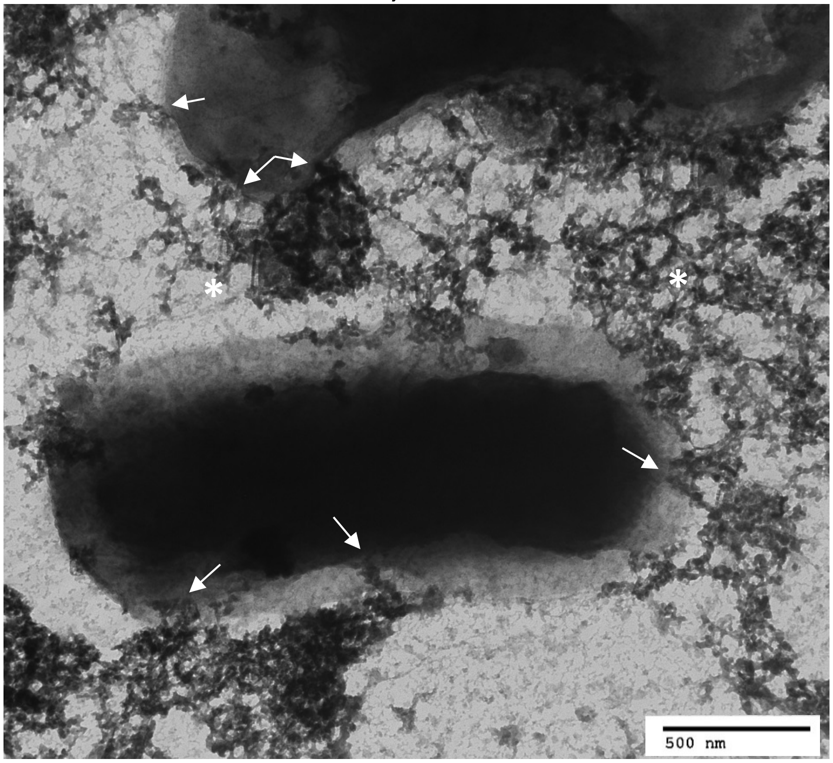

150,000 X

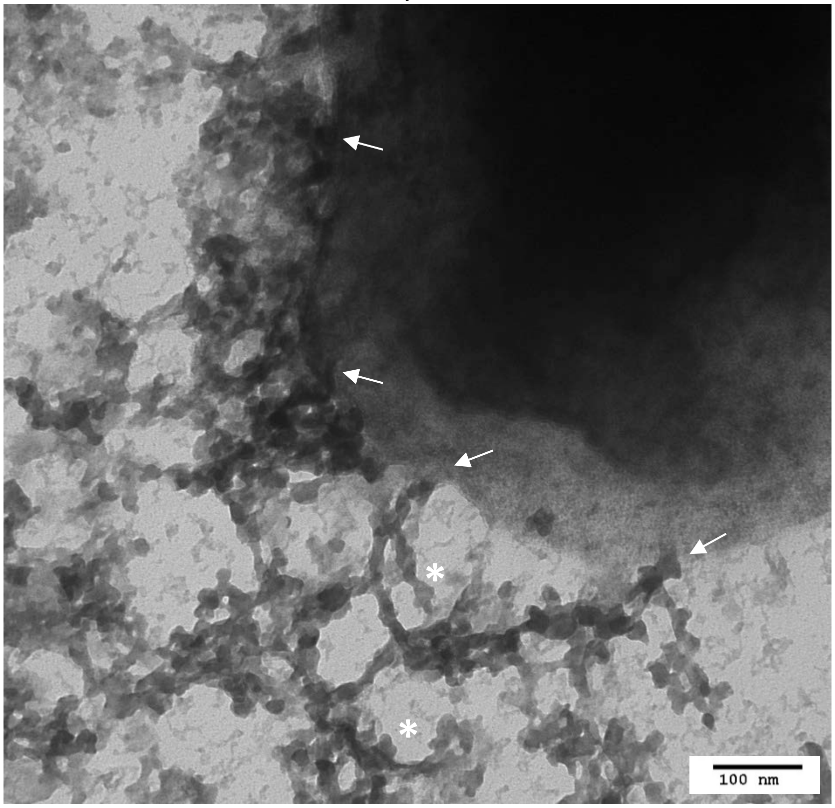

150,000 X

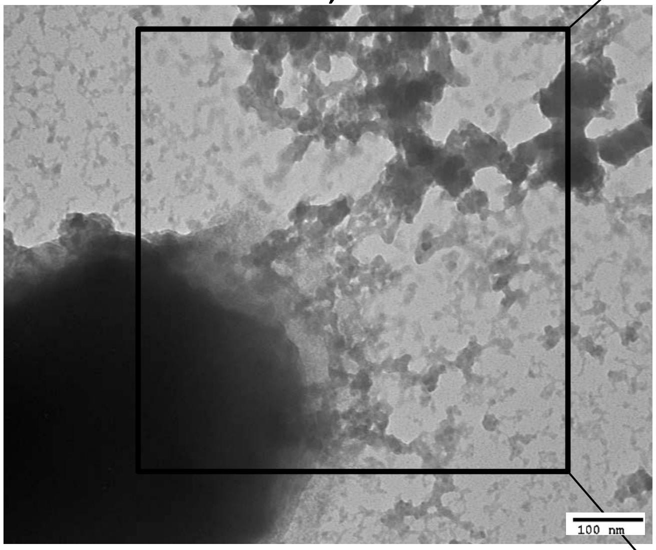

250,000 X

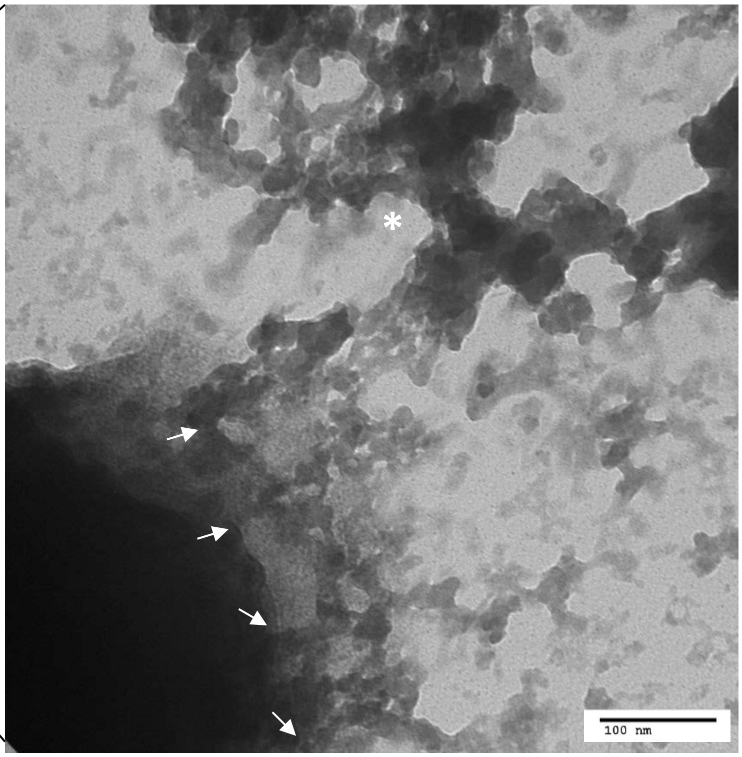

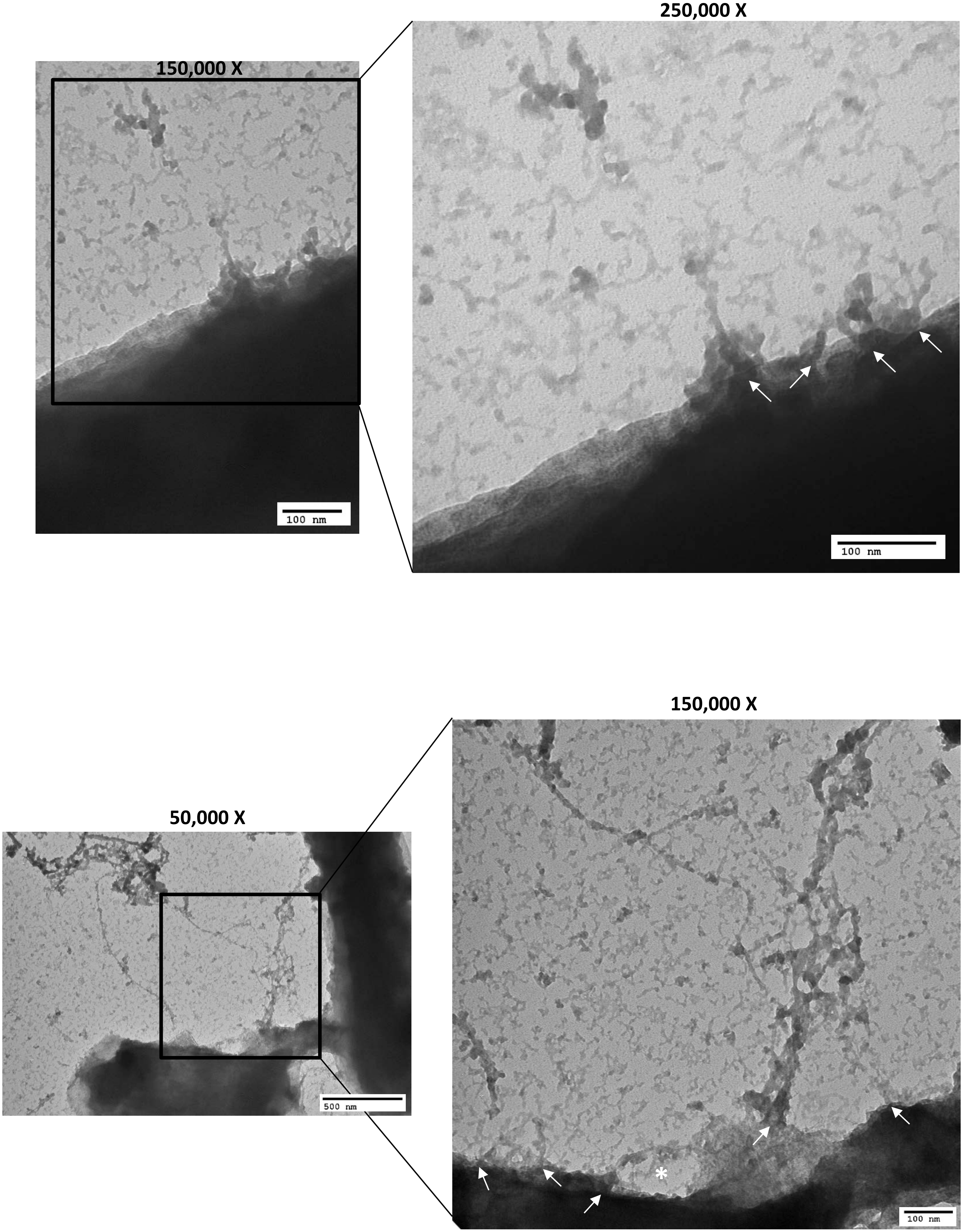
250,000 X
150,000 X
100 nm
100 nm
150,000 X
50,000 X
500 nm
100 nm

B. TSB + Bile Salts

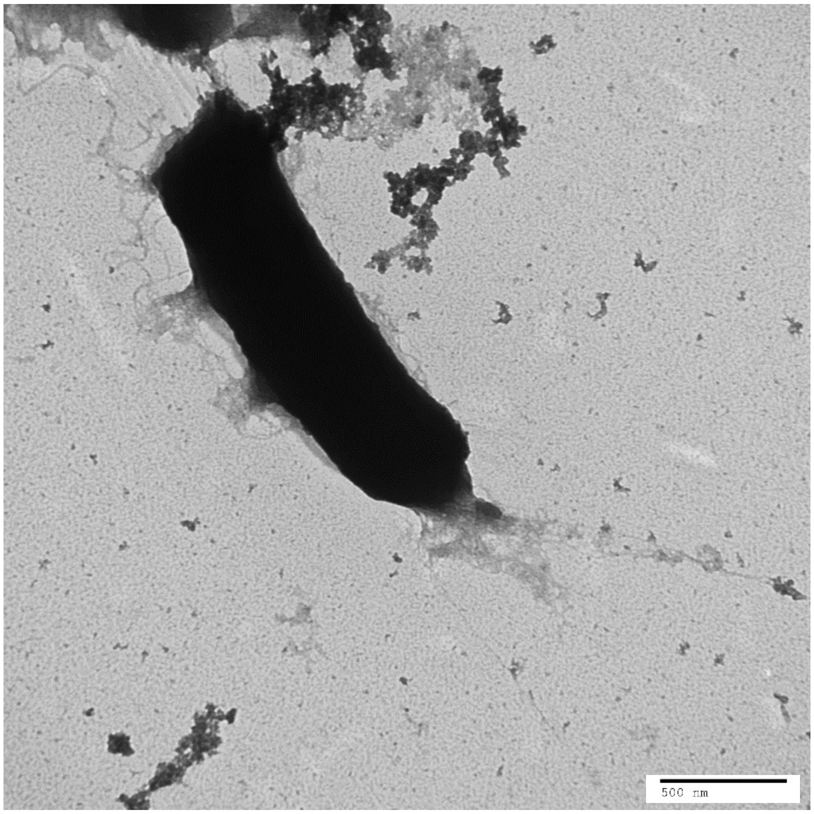

C. Media control images

LB + 2% glucose + 0.4% bile salts

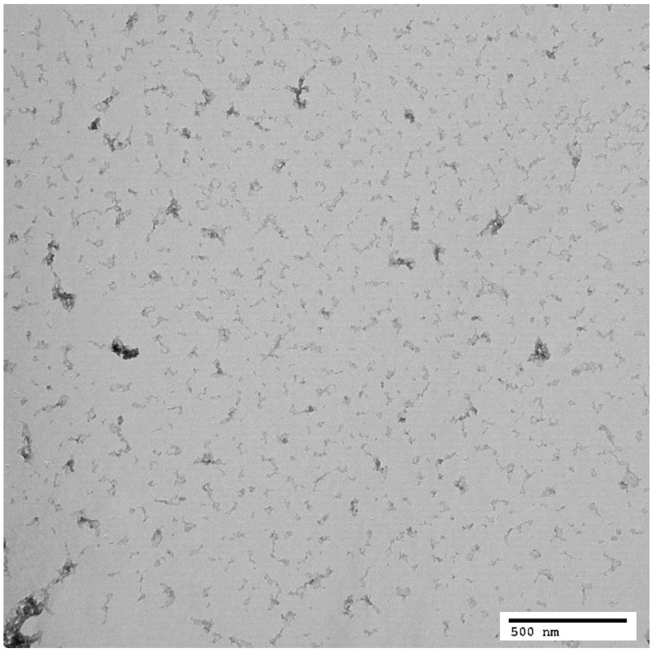

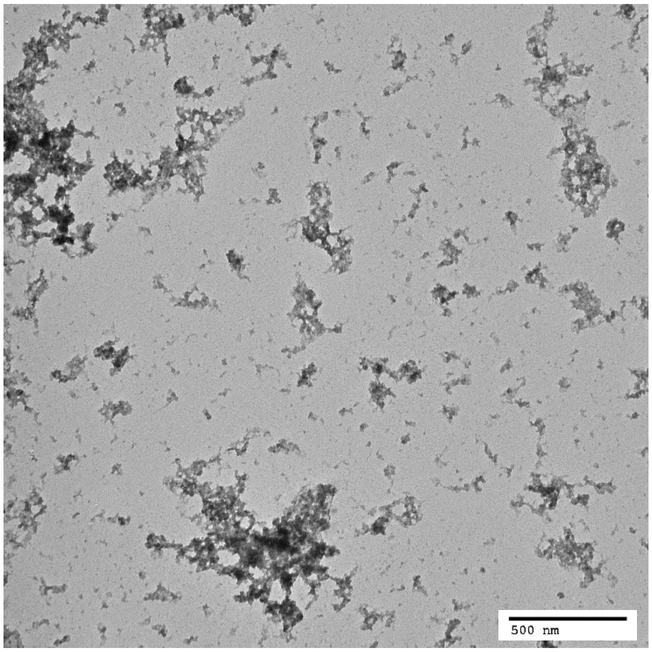

D. *S. flexneri* 2457T IVLC biofilm sample

Area without bacteria

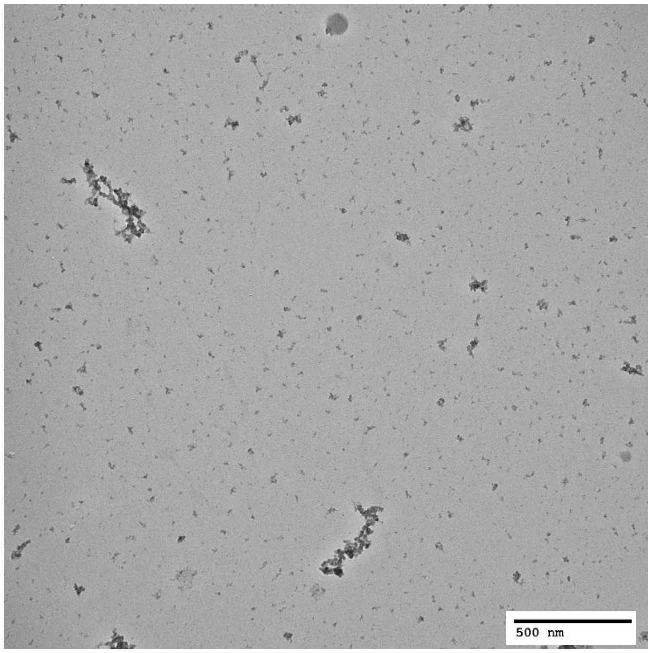

Supplement: FIG S1 [file mSphere.00751-19-sf001.pdf]
