# Supplementary figures and images for: Shigella flexneri Adherence Factor Expression in In Vivo-Like Conditions
Source: mSphere. 2019 Nov 13;4(6):e00751-19. doi: 10.1128/mSphere.00751-19 (PMC6854044; doi:10.1128/mSphere.00751-19)

Supplemental Figure S2

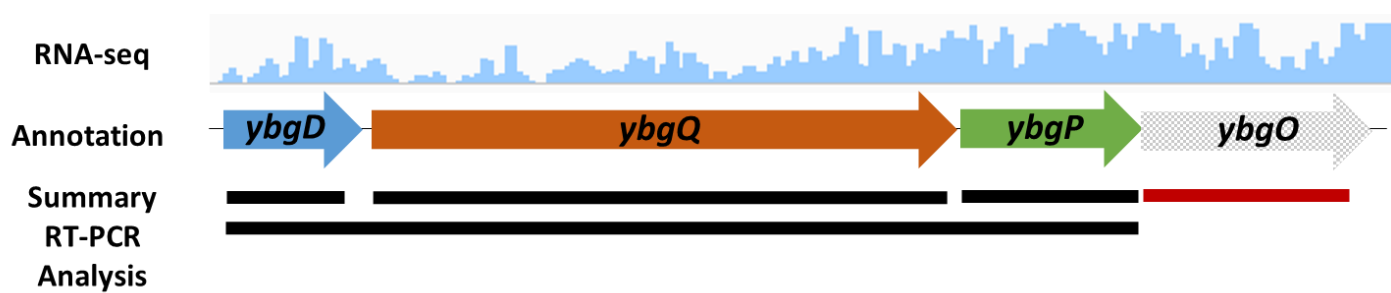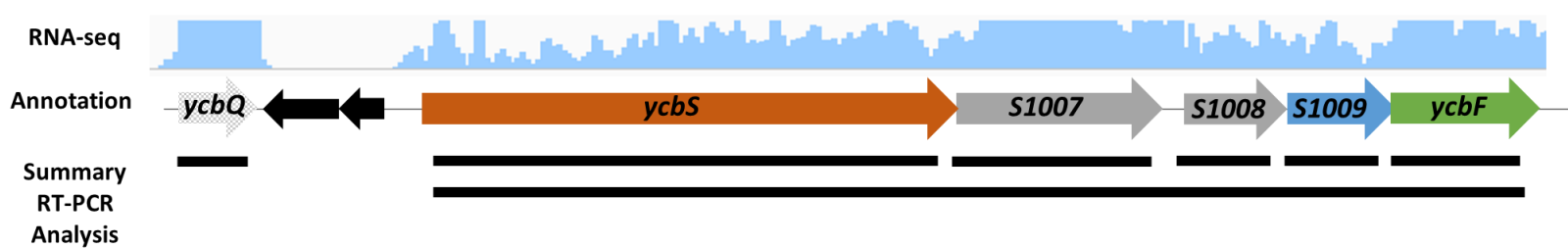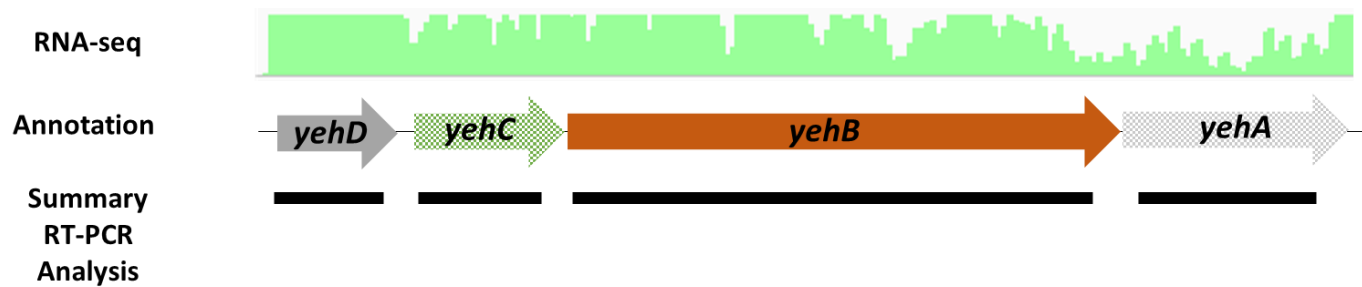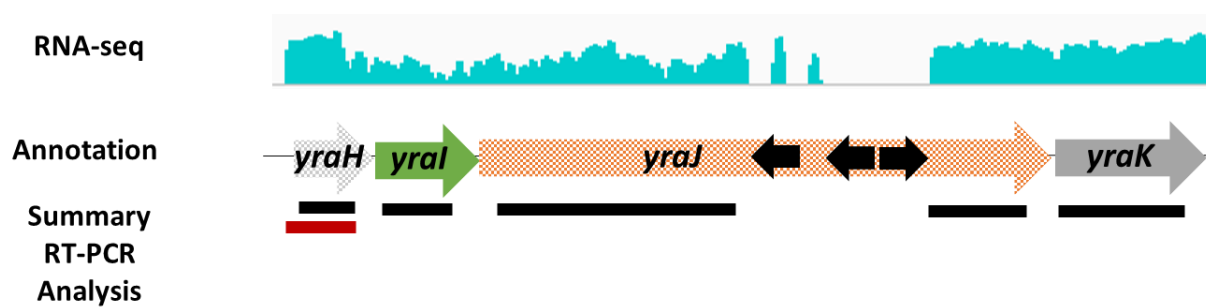

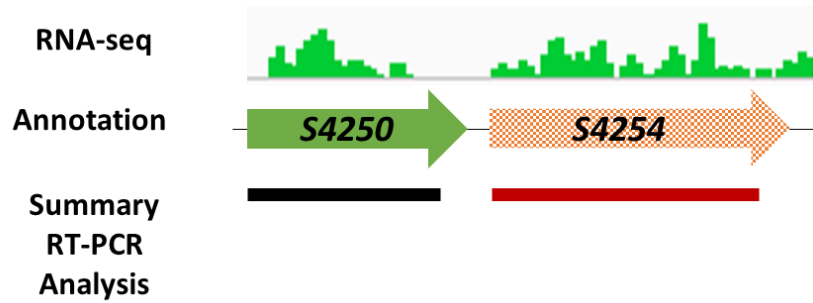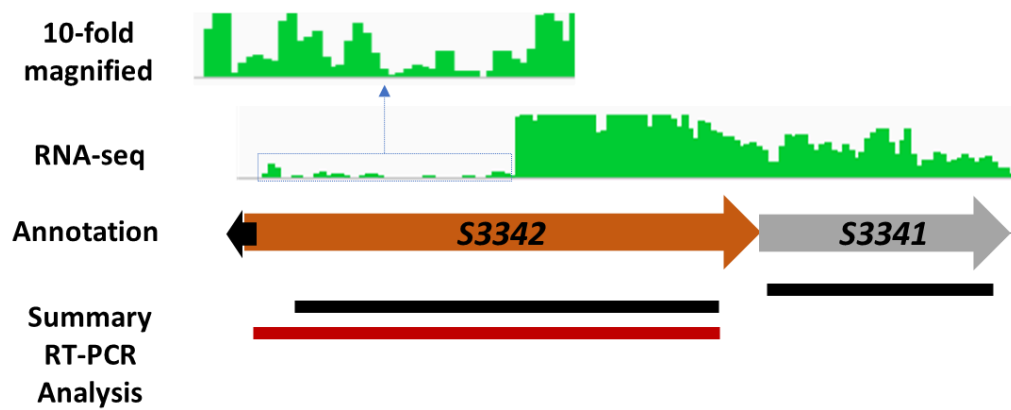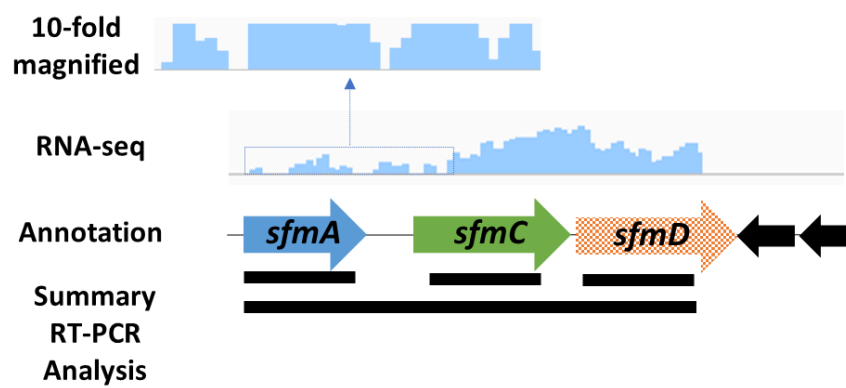

Supplement: FIG S2 [file mSphere.00751-19-sf002.pdf]

Supplemental Figure S3

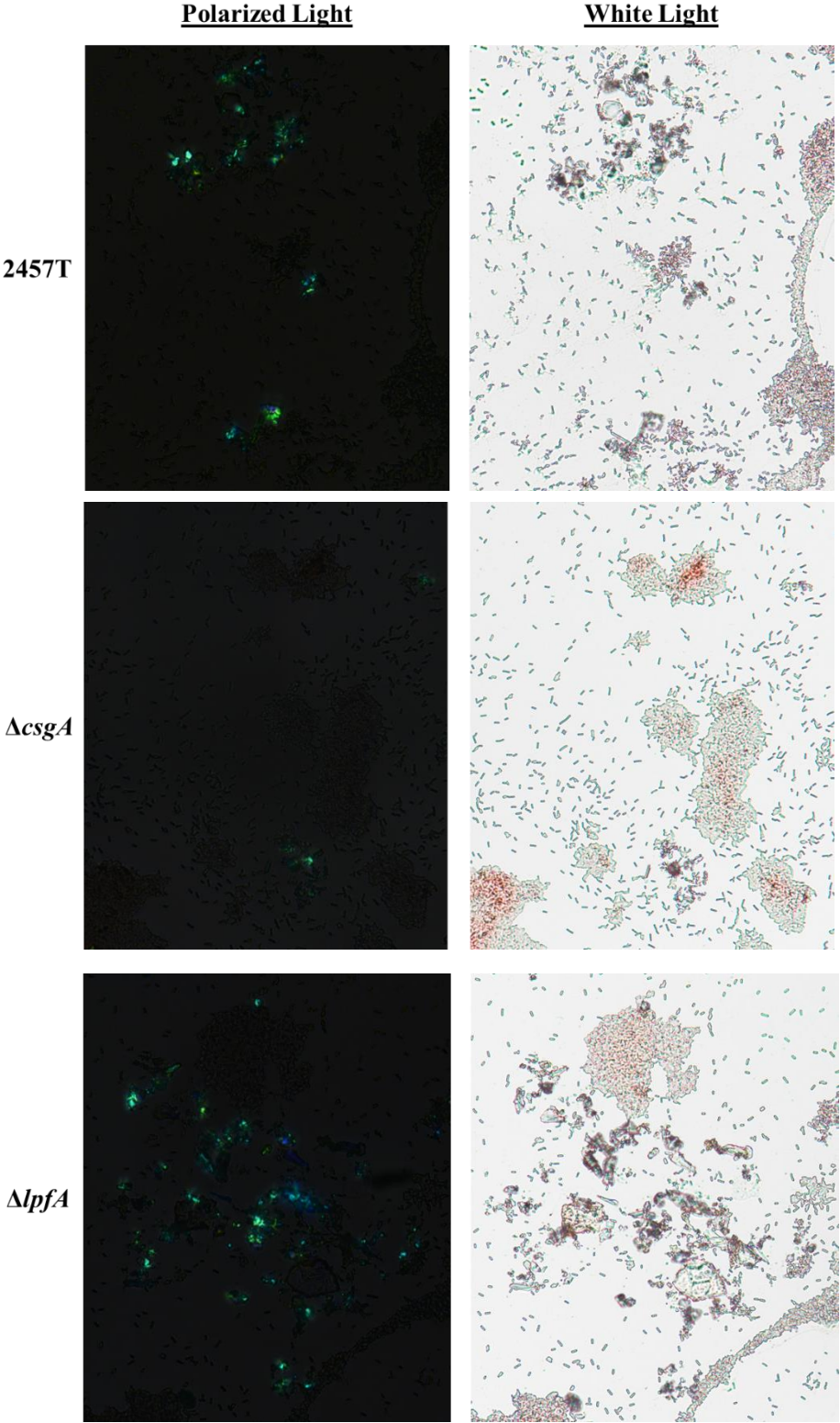

Supplement: FIG S3 [file mSphere.00751-19-sf003.pdf]

Supplemental Figure S4

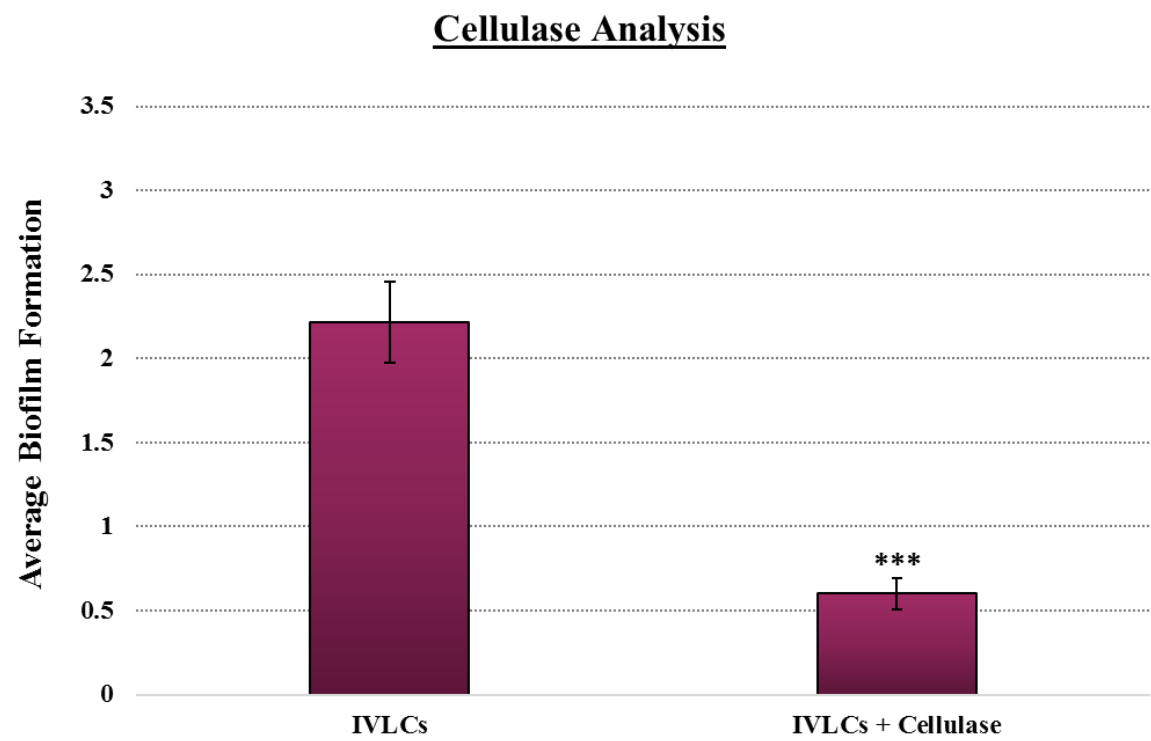

Supplement: FIG S4 [file mSphere.00751-19-sf004.pdf]

Supplemental Figure S5

AF11: *Shigella flexneri* serotype 3a

AF16: *Shigella flexneri* serotype 2a

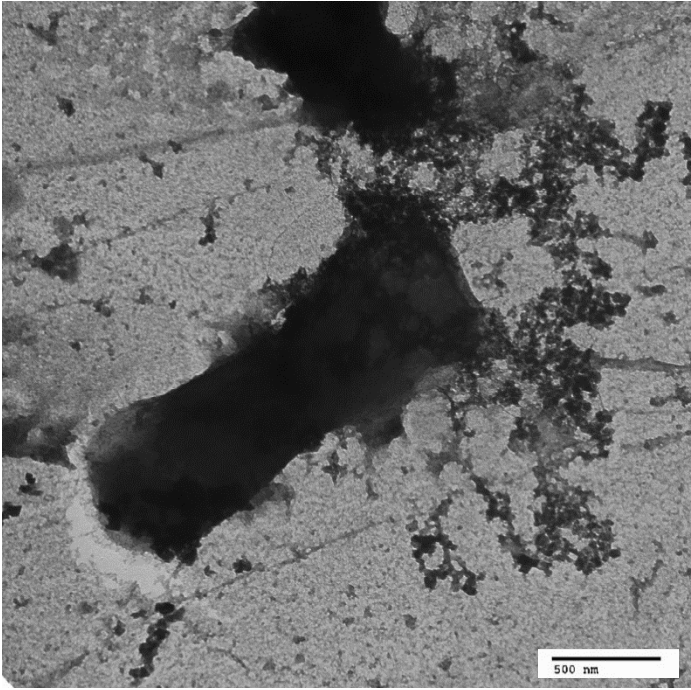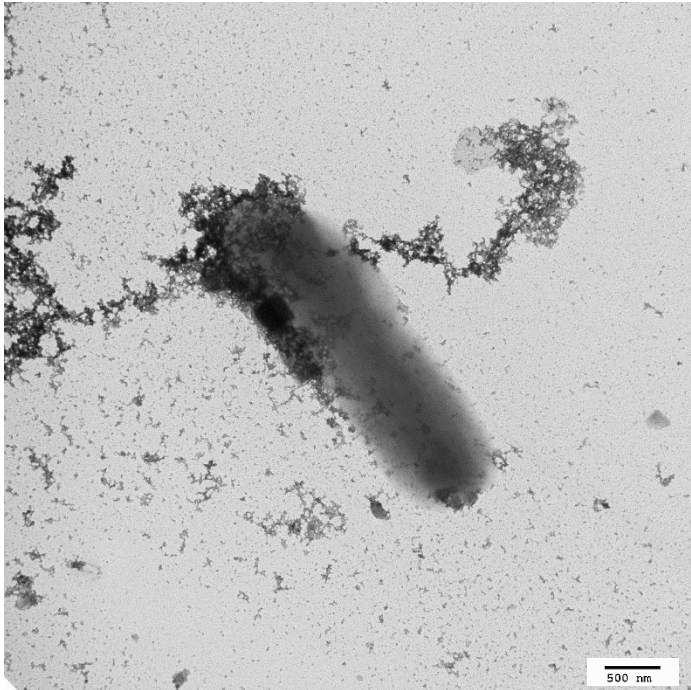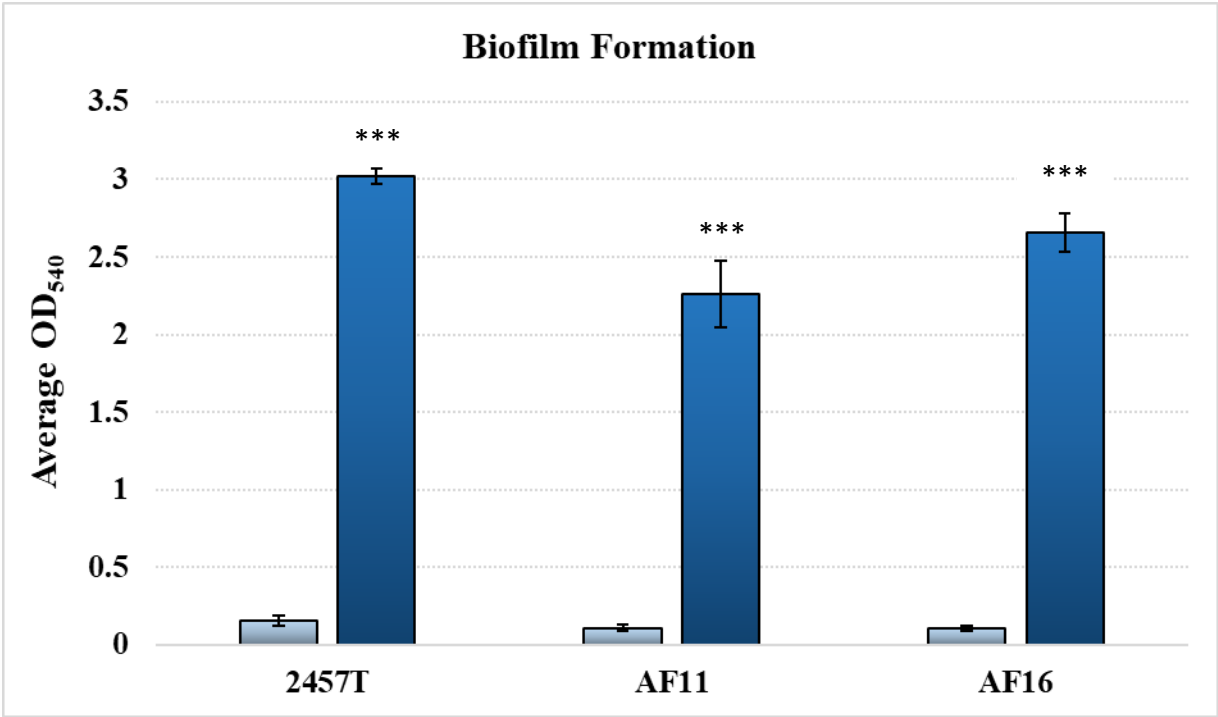

Supplement: FIG S5 [file mSphere.00751-19-sf005.pdf]
